# Bacteria-driven bio-electroactive sterilization

**DOI:** 10.1039/d5sc04234h

**Published:** 2025-07-31

**Authors:** Mingming Qin, Qiuping Qian, Xiaoqing Gao, Tianxi Shen, Feng Jia, Min Wu, Kelong Fan, Yunlong Zhou

**Affiliations:** a Zhejiang Engineering Research Center for Tissue Repair Materials, Wenzhou Institute, University of Chinese Academy of Sciences Wenzhou 325001 P. R. China zhouyl@ucas.ac.cn qianqp@ucas.ac.cn; b Department of Stomatology, Nanchang People's Hospital (The Third Hospital of Nanchang) Nanchang 330009 Jiangxi P. R. China; c CAS Engineering Laboratory for Nanozyme, Key Laboratory of Biomacromolecules (CAS), CAS Center for Excellence in Biomacromolecules, Institute of Biophysics, Chinese Academy of Sciences Beijing 100101 P. R. China

## Abstract

Developing responsive antibacterial materials is crucial in addressing antibiotic overuse. While many materials respond to indirect external stimuli like pH, light, and enzymes, bacterial self-metabolism remains an underutilized activation mechanism for precision sterilization. Here, we present a self-sustaining bioreactor consisting of bacteria-reduced graphene oxide–copper biohybrids (BrGO–Cu), wherein living bacteria activate graphene oxide–copper ions (GO–Cu) for self-termination through metabolic redirection. Bacterial extracellular electron transfer (BEET) cascade reduced graphene oxide promotes Cu^2+^ to Cu^+^ conversion and ultimately kills bacteria through ˙OH generation. Meanwhile, the BrGO–Cu bioreactor effectively prevents biofilm formation with negligible cytotoxicity. Notably, the bacteria-responsive bioreactor exhibits lasting bactericidal activity upon recapture of live bacteria for up to 129 passages without bacterial resistance. Our work pioneers a BEET-redirecting strategy that enables pathogen-specific, long-lasting antimicrobial protection through precisely controlled feedback loops.

## Abstract 2

This BEET-redirecting strategy hijacks bacterial extracellular electron transfer to autonomously drive Cu^2+^ → Cu^+^ conversion and self-amplifying ˙OH cascades, enabling pathogen-specific sterilization without triggering bacterial resistance.

## Graphical abstract

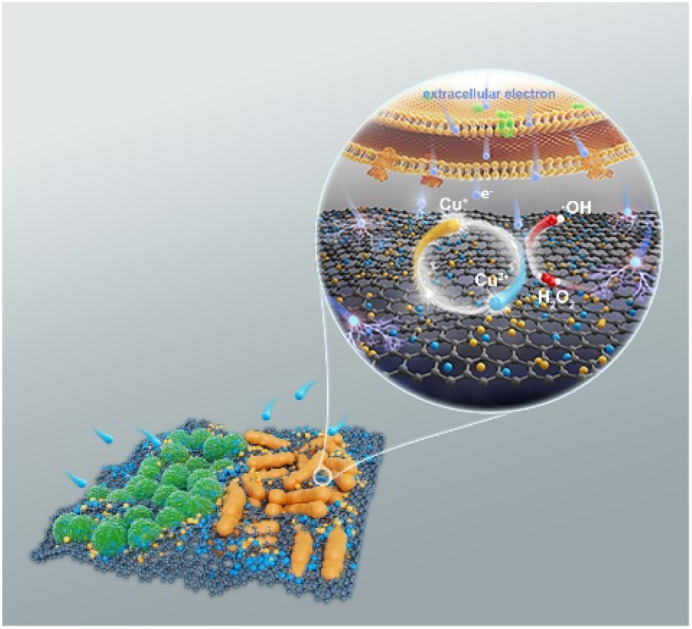

## Introduction

Antibiotics exert therapeutic effects through target-specific molecular interactions; however, this precision inherently imposes evolutionary pressure that accelerates the emergence of resistant pathogens.^1,2^ In contrast, broad-spectrum antimicrobials bypass the limitation of specificity by employing non-selective cytotoxic mechanisms,^3^ primarily through (1) disruption of the cell wall and membrane integrity^4,5^ and (2) ROS-induced peroxidation of lipids, proteins, and DNA.^6,7^ Both mechanisms lead to strong but short-lived antimicrobial effects with potentially severe side effects, as they fundamentally allow uncontrolled continuous release of antimicrobial species. To extend the antimicrobial effect, high doses are inevitable and so is drug resistance. Slow-release systems can delay the development of drug resistance to a certain degree,^8,9^ but on-demand release (*i.e.* stimuli-responsive) is expected to resolve this problem more directly.^10,11^ Secondary chemical or biological cues, such as pH,^12,13^ enzymes,^14^ and light,^15^ are popular choices, but they can be interfered with or mistakenly activated by complicated physiological processes, necessitating novel activation strategies with improved specificity.

Bacterial extracellular electron transfer (BEET), a critical mechanism for energy acquisition and environmental adaptation,^16^ has been increasingly recognized as a contributor to drug tolerance and resistance.^17^ In contrast, eukaryotic cells rely on mitochondrial respiratory chains.^18^ This fundamental distinction positions BEET as a bacteria-specific stimulus, and it is anticipated to be a broad-spectrum and low-side effect ideal method in an antimicrobial scenario. Several studies have demonstrated that blocking BEET induces intracellular oxidative stress, resulting in significant antimicrobial efficacy.^19–22^ Beyond simple inhibition, redirecting BEET to initiate bacteria's own demise is important for on-demand antibacterial effects. Wrapping bacteria on metal-based material forms a responsive and bio-electrochemically active complex, whose antibacterial properties (through the redox reaction) can only be triggered by BEET. This localized and self-activated bactericidal process offers strong potential for minimizing drug dosage, reducing systemic toxicity, and limiting resistance development.

**Fig. 1:**
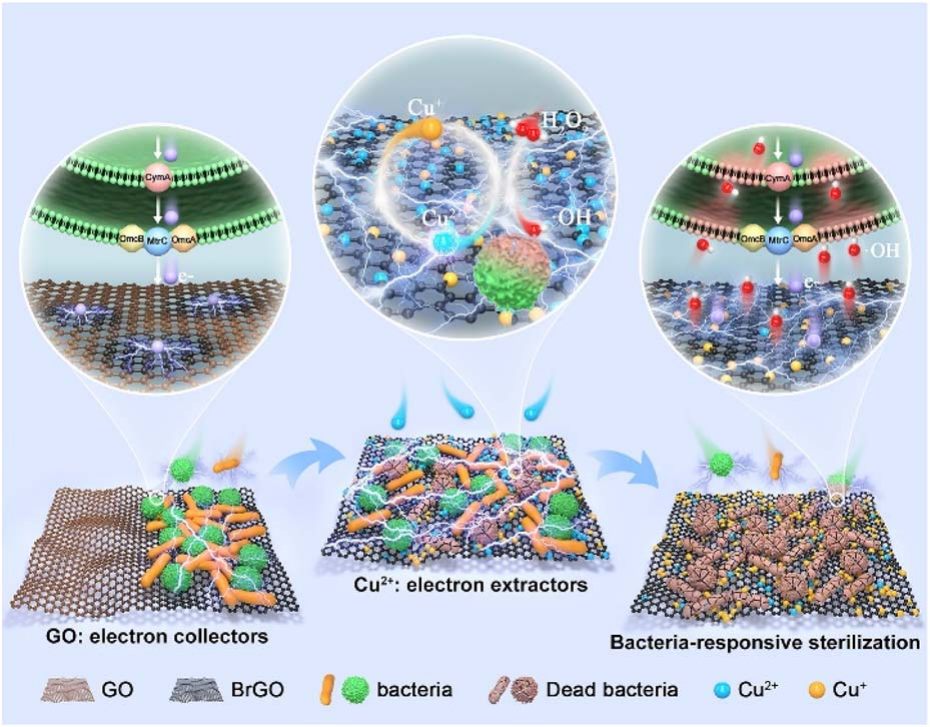
Bio-electroactive sterilization redirects the BEET pathway for *in situ* self-sustaining responsive-sterilization.

Herein, we developed a bio-electroactive sterilization platform through BEET-directed assembly of bacterially reduced graphene oxide with copper ions (BrGO–Cu) for responsive sterilization and long-lasting inhibition of bacterial biofilms (Fig. 1). Graphene oxide (GO) initially captures bacteria through π–π interactions before undergoing bacterial-mediated reduction to BrGO (electron collectors), which subsequently channels extracellular electrons toward Cu^2+^ centers (electron extractors). This bioelectrochemical configuration redirects the BEET pathway to amplify the Cu^2+^/Cu^+^ redox cycle and depletes glutathione (GSH) within biofilms, promoting a Fenton-like reaction that disrupts bacterial structures. Simultaneously, BrGO hijacks BEET to generate H_2_O_2_*in situ* for self-sustained antibiofilm efficacy.

## Results and discussion

### Fabrication and characterization of the BrGO–Cu bioreactor

The BrGO–Cu bioreactor was prepared in two steps (Fig. 1). First, the BrGO biohybrids formed as living bacteria's outer lipids adhered to the amphiphilic GO surface.^23^ Subsequently, a copper chloride (CuCl_2_, Cu^2+^) solution was introduced to form BrGO–Cu biohybrids *via* cation–π interaction.^24^ The bioreactor consists of two functional units: an electron collector (BrGO biohybrid) and an electron extractor (catalytically active site, Cu ions) for a Fenton-like reaction through valence-modulation later.

**Fig. 2:**
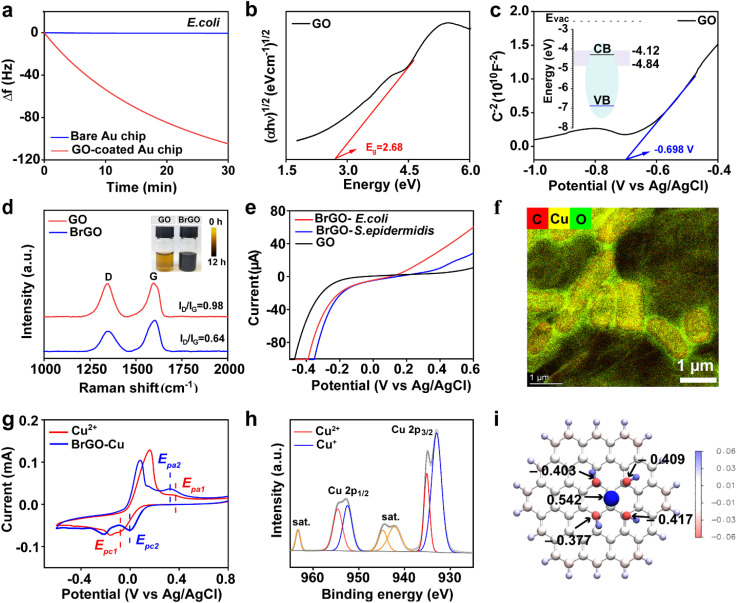
(a) Frequency changes of *E. coli* adsorption on bare and GO-coated Au chips. (b) Tauc's plots and (c) Mott–Schottky plots of GO; the inset in (c) shows the energy levels of GO. The light pink area indicates biological redox potential range. (d) Raman spectra and the corresponding color (inset) of GO and BrGO (*I*_D_/*I*_G_: intensity ratio of the D and G bands). (e) Bacterial extracellular electron transfer from the bacteria to GO detected using the *I*–*V* curves. (f) Element mapping of Cu in the BrGO–Cu bioreactor. (g) CV curves before and after the assembly of Cu^2+^ and BrGO hybrids. (h) XPS spectra of Cu 2p in the BrGO–Cu bioreactor. (i) The relevant ADCH charge distribution of rGO–Cu^2+^ (C: cyan, O: red, Cu: orange, and H: white).

To efficiently gather bacterial extracellular electrons (BEEs), the electron collector should be constructed from a material possessing good water dispersibility, strong bacterial adhesion, and the redox potential below the biological redox potential (BRP), such as two-dimensional GO nanosheets. The as-obtained GO disperses well in water, with an average zeta potential of −51 ± 1 mV, as depicted in Fig. S1. This excellent dispersion stability is attributed to the presence of carboxyl (–COOH) groups primarily located at the edges of the film, ensuring good contact with bacteria (Fig. S1, FTIR spectra). GO's hydrophobic basal plane (evidenced by the C–C/C=C peaks in XPS, Fig. S2) provides strong adhesion to bacteria through hydrophobic interaction with bacterial lipids.^25^ The ultrahigh affinity between GO and bacteria (*E. coli* and *S. epidermidis*) was demonstrated through time-dependent bacterial adherence behavior analysis using a quartz crystal microbalance with dissipation (QCM-D). Notable time-dependent frequency changes were observed when the bacterial suspension adhered to the GO-coated QCM crystal, indicating rapid bacterial capture by GO (Fig. 2a and S3). SEM analysis further confirmed that GO efficiently captured multiple live bacteria, suggesting that bacteria could serve as an electron supply source through BEET (Fig. S4 and S5). Successful electron transfer from bacteria to the electron collector (BrGO) is the key for BEET stimulated sterilization, which is related to the redox potential of GO. Ultraviolet-visible (UV-vis) spectroscopy and Mott–Schottky (M–S) electrochemical testing were further employed to investigate the energy level of GO in detail. Tauc's plots converted from UV-vis spectra revealed a bandgap energy (*E*_g_) of 2.68 eV for GO (Fig. S6 and 2b).^26^ Furthermore, GO is an n-type semiconductor as observed from the positive spectral slope from the M–S spectra, with a −0.698 V (*vs.* Ag/AgCl) flat band potential (*E*_fb_) (Fig. 2c).^27^ The potential applied to Ag/AgCl was converted to the reversible hydrogen electrode (RHE) potential using formula (1):1
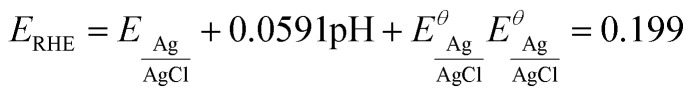


Therefore, the flat band potential is −0.0853 V (*vs.* RHE), indicating that the minimum conduction band potential (*E*_CB_) was −0.1853 V (*vs.* RHE) calculated from the empirical formula (*E*_CB_ = *E*_fb_ − 0.1, where 0.1 is an empirical value). The RHE potential can be converted to the energy level position under vacuum using eqn (2):

2 *E*_*vs.* vacuum_ = −4.5 − *E*_RHE_

Consequently, the corresponding *E*_CB_ at the vacuum level was determined to be −4.3147 eV (*versus* vacuum). According to the equation *E*_g_ = *E*_VB_ − *E*_CB_, the valence band (VB) position is −6.9947 eV (*versus* vacuum), in good agreement with previous reports. Bacteria typically possess a biological redox potential (BRP) ranging from −4.12 to −4.84 eV due to the membrane disulfide bonds,^28,29^ higher than the potential of GO. This ensures the rapid transfer of electrons from bacterial membrane proteins to GO. The evidence of directing BEET to GO was obtained using Raman spectroscopy (Fig. 2d and S7). After co-culturing with bacteria for 12 h, the (*I*_D_/*I*_G_) ratio decreased from 0.98 to 0.64, indicating a significantly higher graphitization degree due to BEET-mediated reduction.^30–32^ The electrons collected from bacteria were measured from the current–potential (*I*–*V*) curve. As shown in Fig. 2e, a more saturated current was observed in the BrGO group cultured with *E. coli* (Gram-negative bacteria, G−) than *S. epidermidis* (Gram-positive bacteria, G+), as a consequence of the thicker cell wall in *S. epidermidis* and thus a lower BEET rate. Moreover, electrochemical impedance spectroscopy (EIS) showed that the resistance of the BrGO was *ca.* 13.5 Ω, four times lower than that of the pure GO film (*ca.* 67 Ω; Fig. S8). This lower electrochemical impedance suggests a stronger electron transport capacity for BrGO compared to GO, beneficial for the electron extraction later.

The stable binding between Cu ions and BrGO is crucial for shortening the electron transfer path and promoting rapid electron extraction. Energy-dispersive spectroscopy (EDS) was first performed to explore the Cu element distribution in the bioreactor. As depicted in Fig. 2f and S9, the Cu element was mainly distributed at the interface of bacteria and BrGO. After washing with deionized-water two times, the Cu ion concentration desorbed from BrGO–Cu was much lower than 1.5 μM (Fig. S10), indicating that over 95% of Cu ions were stably bound to BrGO. To further investigate the electron extraction capacity of Cu ions, a series of experiments were conducted. Cyclic voltammetry (CV) was subsequently conducted to explore electron extraction from BrGO by Cu^2+^ (Fig. 2g and S11). Both the anodic and cathodic peak potentials in the CV curve of BrGO–Cu were shifted to the middle voltage window compared to Cu^2+^ alone, demonstrating that the reductive ability of BrGO–Cu was much higher than Cu^2+^ possibly as a result of assigning too many electrons to Cu^2+^ for the BrGO biohybrid (see Table S1 for details). XPS was performed to investigate the oxidation states of Cu ions in the BrGO–Cu bioreactor as an indicator of electron extraction. The binding energy of the Cu 2p spectrum, as indicated in Fig. 2h, shifted from 934.7 eV (Cu 2p_3/2_) and 954.7 eV (Cu 2p_1/2_) to 932.3 eV (Cu 2p_3/2_) and 952.2 eV (Cu 2p_1/2_), respectively, revealing the presence of Cu^2+^ and Cu^+^ valence states in the BrGO–Cu bioreactor and a successful electron extraction from the BrGO electron collectors. To further elucidate the electron extraction behaviour of Cu in the BrGO biohybrid, density functional theory (DFT) calculations were performed (see SI, Experimental procedures).^33^ As illustrated in Fig. S12, the highest occupied states of the molecular orbitals (HOMO) exhibit strong coupling, namely cation–π interaction, between the empty d orbitals of Cu^2+^ and delocalized π orbitals of the aromatic structure of the BrGO surface. This interaction as the electron transfer path resulted in a noticeable charge transfer from BrGO to Cu^2+^. The atomic dipole moment corrected Hirshfeld population (ADCH) charge distributions showed that Cu^2+^ on BrGO retained a partial charge of +0.542 compared to the original +2 valence, further confirming the significant charge transfer between BrGO and Cu^2+^ (Fig. 2i).

### *In situ* living bacteria-driven antibiofilm performance

**Fig. 3:**
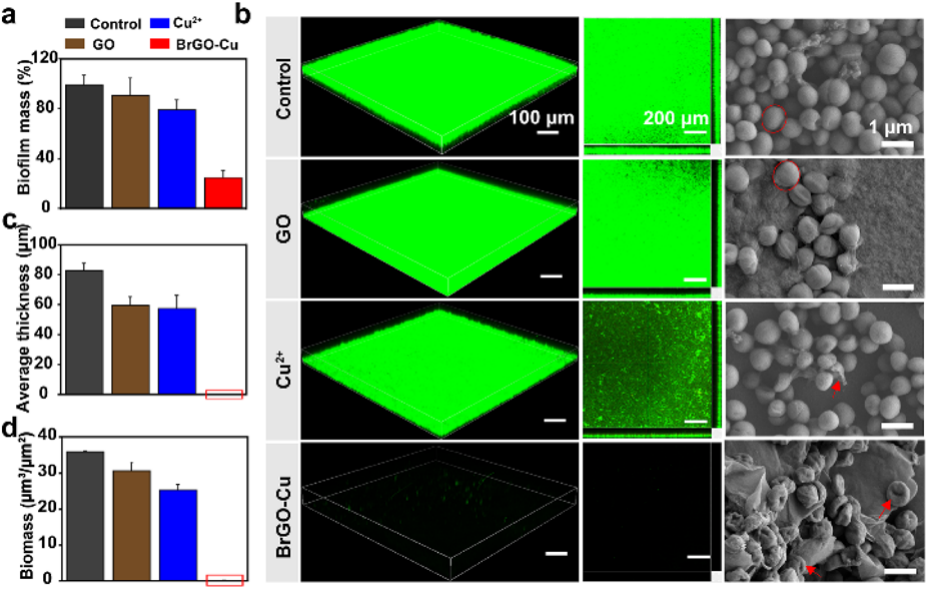
(a) Histograms of *S. epidermidis* exposed to stroke-physiological saline solution (SPSS; control), GO (125 μg mL^−1^), Cu^2+^ (100 μM), and BrGO–Cu (containing 125 μg mL^−1^ GO and 100 μM Cu^2+^) biohybrids for 72 h. (b) CLSM and SEM images of *S. epidermidis* biofilms treated with SPSS, Cu^2+^, GO, and BrGO–Cu biohybrids for 72 h (green fluorescence: live *S. epidermidis* biofilm). Average thickness (c) and (d) biomass of *S. epidermidis* biofilms derived from COMSTAT analysis of CLSM images.

Due to its high redox potential, Cu^+^ efficiently catalyzes trace hydrogen peroxide (H_2_O_2_) in bacterial environments to generate reactive oxygen species (ROS),^34,35^ killing bacteria and inhibiting biofilm formation. *S. epidermidis* and *E. coli* were incubated with different concentrations of GO, Cu^2+^, and BrGO–Cu. As shown in Fig. 3a, neither Cu^2+^ nor GO had an obvious inhibitory effect on bacterial biofilm formation. The increase in biofilm mass with increasing GO concentration is attributed to GO sheets absorbing nutrients to form protein corona, significantly promoting rapid bacterial proliferation. While the antibiofilm activity of Cu^2+^ increased in a concentration-dependent manner (Fig. S13), the inhibition efficacy could only reach about 25% at 100 μM. However, the biofilm inhibition efficiency was boosted up to 75% when Cu^2+^ (100 μM) was introduced into the co-culture of bacteria and GO (125 μg mL^−1^).

To further verify the antibiofilm potency of the BrGO–Cu bioreactor, confocal laser scanning microscopy (CLSM) and electron scanning microscopy (SEM) were performed (Fig. 3b). In the control group, the cell wall was intact and smooth, forming a thick and dense biofilm after saline physiological water (SPPS) treatment. In contrast, in the GO and Cu^2+^ groups, biofilm formation was slightly inhibited, with some cell walls showing slight shrinkage or rupture, presumably due to the elevated ROS levels and transient nutrient isolation, respectively. The BrGO–Cu bioreactor causes severe cell wall damage and almost completely shuts down bacterial biofilm formation, achieving maximum anti-biofilm efficacy. Moreover, COMSTAT analysis revealed that the *S. epidermidis* biofilm increased to 85 μm thick after treatment with SPSS. Conversely, the biofilm thickness was close to 0 μm in the BrGO–Cu group (Fig. 3c). In the Cu^2+^ and GO groups, the biofilm thickness was comparable, but the biomass was significantly higher in the GO group, further indicating that GO had no anti-biofilm effect. Obviously, the biomass approached 0 μm^3^ μm^−2^ in the BrGO–Cu bioreactor, with the bacteria largely suicidal, effectively preventing biofilm formation (Fig. 3d). Similar conclusions were reached in experiments with *E. coli* and *MRSA* (Fig. S14–S16). Taken together, although GO films capture a large number of bacteria to prevent the escape of free bacteria and inhibit bacterial reproduction for a short time through nutrient isolation, it has no obvious killing effect on bacteria. The enrichment of living bacteria actively generates abundant extracellular electrons, promoting Cu^2+^ to Cu^+^ conversion. Within the enriched live bacteria, the BrGO–Cu bioreactor redirected BEET to power Cu-mediated apoptosis, thus effectively inhibiting bacterial biofilm formation on substrate surfaces.

### Bacteria-responsive antimicrobial performance

**Fig. 4:**
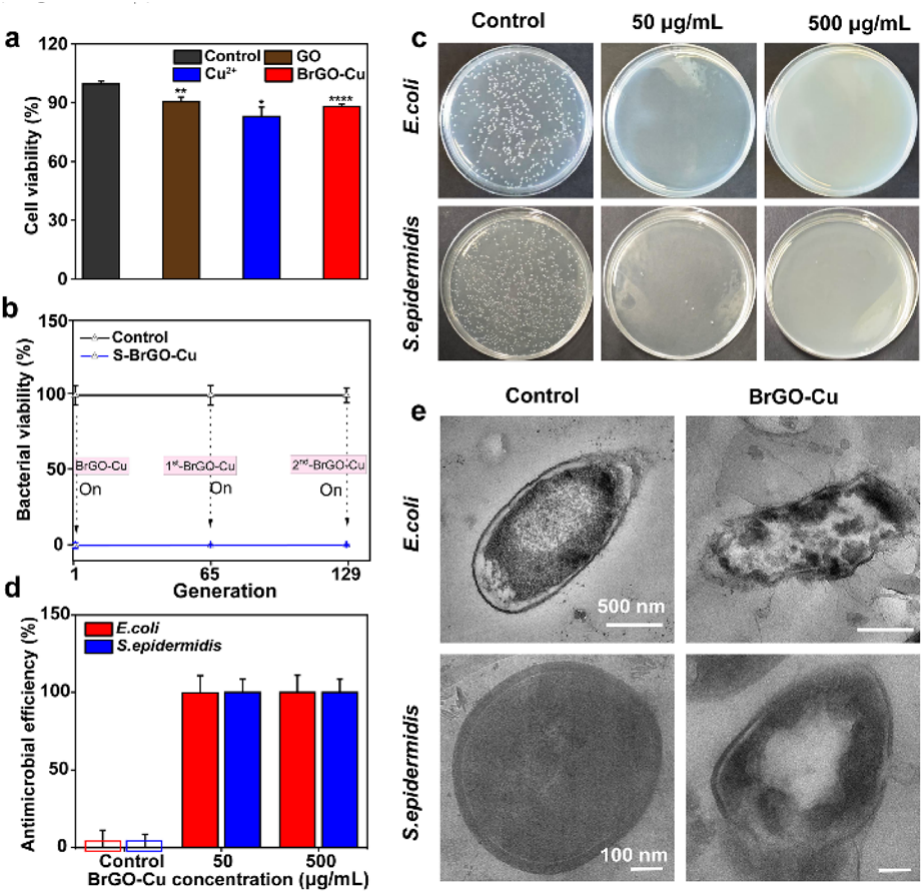
(a) Cell toxicity evaluation of stroke-physiological saline solution (SPSS, control), GO, Cu^2+^, and the BrGO–Cu bioreactor on L929 cells at 37 °C for 24 h. (b) Bacterial viability following successive introductions of various generations into the same BrGO–Cu bioreactor. (c) Photographs, (d) quantitative statistical results and (e) TEM images with the inactivated BrGO–Cu bioreactor.

The precise bacteria-responsive antimicrobial bioreactors were highly specific leaving normal tissues untouched. The *in vitro* biocompatibility of GO, Cu^2+^, and BrGO–Cu bioreactors was investigated by examining their toxicity on the L929 cell line. After treatment with GO, Cu^2+^, and BrGO–Cu bioreactors at 125 μg mL^−1^, 100 μM and 125 μg mL^−1^ to 100 μM separately, the survival rates of the cells were 92.8%, 84.4% and 88.0% (Fig. 4a). Furthermore, hemolysis assessment of bioreactors shows a hemolysis rate of less than 5.0% at concentrations up to 125 μg mL^−1^ to 100 μM, indicating good hemocompatibility (Fig. S16).

Furthermore, BrGO–Cu turned on antimicrobial processes only in the presence of living bacteria, a crucial step in preventing resistance development. To rigorously evaluate this, we conducted a long-term resistance assessment by continuously culturing bacteria under sublethal BrGO–Cu exposure (1/2 MBC) for up to 129 generations. As shown in Fig. 4b, the 65th generation of *S. epidermidis* was completely eradicated upon co-culture with the 1st-BrGO–Cu bioreactor (125 μg mL^−1^ to 100 μM). Re-introducing the 129th generation bacteria into the system, 2nd-BrGO–Cu could be rapidly reactivated for sterilization, confirming its excellent bacteria-responsive antimicrobial activity. The same principle also applied to the inactivation of *E. coli* by the BrGO–Cu bioreactor (Fig. S17). In addition, sublethal concentrations of BrGO–Cu treated strains did not change the effective antimicrobial concentration, further suggesting that the BrGO–Cu bioreactor curtails the generation of drug-resistant bacteria. These results showed that the bioreactors did not exhibit significant toxicity at 125 μg mL^−1^ to 100 μM, but rather effectively in bacteria-responsive sterilization. It is also worth noting that after three sterilization cycles, the concentrations of copper ions released into the surrounding solution in the BrGO–Cu bioreactor solution remained at approximately 0.1 μM (Fig. S18), indicating minimal ion release and strong copper ion retention. However, the accumulation of bacterial debris during this process may increase internal resistance, hinder electron transfer,^36^ and lead to a decline in bacterial current within the bioreactor (Fig. S19), suggesting the need for surface optimization to maintain long-term performance.

The final proof-of-concept tested whether inactivated BrGO–Cu could still trigger bacteria-responsive antimicrobial activity as a reagent. As shown in Fig. 4c and d, the inactivated BrGO–Cu displayed significant bacteria-responsive behavior at a concentration of 50 μg mL^−1^, achieving >99% antimicrobial efficiency against both *E. coli* and *S. epidermidis* after 2-hour exposure. Furthermore, significant cytoplasmic loss and/or bacterial degradation were observed *via* TEM imaging in the BrGO–Cu treated group compared to the control (Fig. 4e). The treatment with inactivated BrGO–Cu induced bacterial cell wall membrane shrinkage and/or structural damage, resulting in cytoplasmic leakage, confirming the potential of inactivated BrGO–Cu as an excellent bacteria-responsive antimicrobial agent.

### BrGO–Cu bioreactor redirects BEET to enhance the catalytic antibiofilm mechanism

**Fig. 5:**
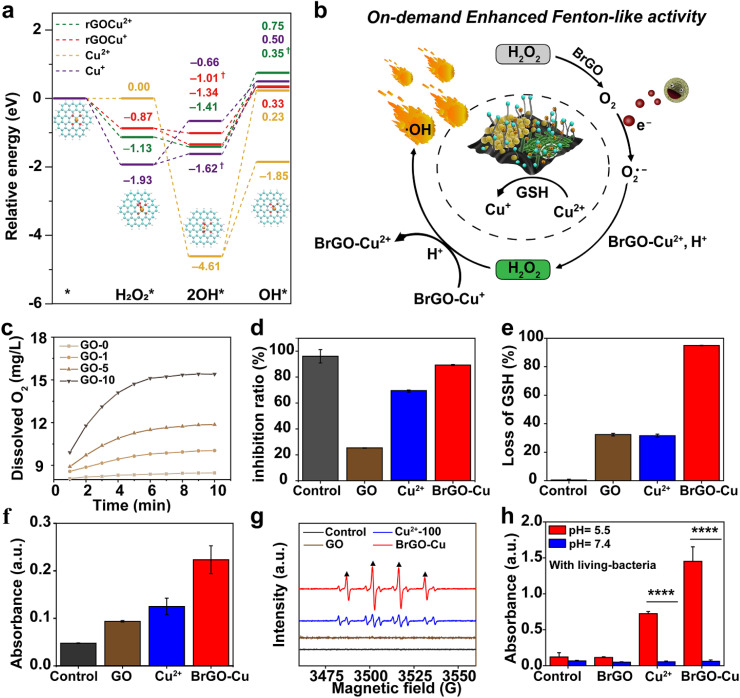
(a) Proposed catalytic mechanism schematic and the free energy diagrams of Cu^+^, Cu^2+^, rGO–Cu^2+^, and rGO–Cu^+^ in the Fenton-like process (C: cyan, O: red, Cu: orange, and H: white). (b) Diagram of the process of the enhanced valence regulation process by BEET-driven redox species. (c) O_2_ production ability of different concentrations of GO by catalysing H_2_O_2_ in a bacterial fluid. (d) O_2_˙^−^ scavenging activities of SOD enzymes (control), GO (125 μg mL^−1^), Cu^2+^ (100 μM), and BrGO–Cu (containing 125 μg mL^−1^ GO and 100 μM Cu^2+^). (e) Loss of GSH (%) after 10 h incubation with the different components. (f) H_2_O_2_ generation abilities of living bacteria, GO, Cu^2+^ and BrGO–Cu. (g) ESR spectra for Fenton reaction-induced ˙OH generation in the biofilm microenvironment in the presence of H_2_O_2_. (h) ˙OH generation activity *via* TMB-based UV-vis spectra in biofilm microenvironment pH (∼5.5) and physiological pH (∼7.4) in the presence of H_2_O_2_ (0.1 mM).

Typically, Cu-based materials could decompose H_2_O_2_ into hydroxyl radicals (˙OH) within the biofilm microenvironment, effectively killing bacteria by oxidizing proteins, lipids, and nucleic acids.^37–39^ To investigate the mechanism of redirected BEET-activated Cu ion valence modulation for enhanced antibiofilm properties, we conducted DFT analysis on H_2_O_2_ decomposition by the BrGO–Cu bioreactor. The process of catalyzing H_2_O_2_ is shown in Fig. 5a, including three steps: adsorption, homolysis, and desorption (BrGO–Cu^*x*+^ + H_2_O_2_ → BrGO–Cu^*x*+^·H_2_O → BrGO–Cu^2*x*+^˙OH + OH, *x*+: oxidation states; the main rGO–Cu was taken as the calculation model).^40^ The desorption processes of free radicals from rGO–Cu^2+^, rGO–Cu^+^, Cu^+^, and Cu^2+^ are endothermic, which is the rate-determining step of the whole reaction. However, the desorption activation energy of rGO–Cu^+^ was only 1.67 eV. The activation energies of the entire reaction were 1.76, 1.67, 2.43, and 4.84 eV on rGO–Cu^2+^, rGO–Cu^+^, Cu^+^, and Cu^2+^, respectively (Table S2). rGO–Cu^2+^, compared to Cu^2+^, has significantly lower energy to overcome in the rate-determining step, suggesting that Cu^+^ is more favorable to catalyze the generation of ˙OH from H_2_O_2_. Moreover, the rGO–Cu system inhibited biofilm formation less effectively than the BrGO–Cu bioreactor (Fig. S20), likely due to its lower Cu^+^ content. The Cu^+^/Cu^2+^ ratio, calculated from the XPS Cu 2p spectra (Fig. S21), was in the order of BrGO–Cu > rGO–Cu > bacteria–Cu > GO–Cu^2+^. This implies that the valence modulation of the BrGO–Cu bioreactor depends on redirected BEET-activated chemical units with potential turbulence from O_2_, H_2_O_2_ and GSH in the biofilm microenvironment (BME, Fig. 5b).^41,42^

As shown in Fig. 5c, the BrGO effectively catalyzes H_2_O_2_ to O_2_. Bacteria in the bacterial biofilm were in oxide-limited BME with abundant electron donors and few electron acceptors;^43^ thus, O_2_ would be easily reduced to superoxide anion radicals (O_2_˙^−^) by BEE.^44^ Both Cu and BrGO–Cu display a consistently higher O_2_˙^−^ elimination capacity than pure BrGO as expected, indicating that Cu ions were the main component of scavenging O_2_˙^−^ (Fig. 5d). Moreover, the BrGO–Cu (+2/+1) system can effectively consume the GSH in the biofilms (Fig. 5e). With the sequential addition of BrGO and Cu^2+^, the characteristic absorbance decreased, effectively depleting GSH. Therefore, the increased ratio of Cu^+^/Cu^2+^ resulted from the simultaneous presence of O_2_˙^−^ and GSH, which was consistent with the Cu 2p XPS results (Fig. S21). Additionally, BrGO–Cu can be transformed into BrGO–Cu^+^ and H_2_O_2_ in the subacid BME. The generation of H_2_O_2_ was monitored by its indicator Ti(SO_4_)_2_, which turned yellow with a characteristic absorption peak at 415 nm. Fig. 5f shows a considerable increase in the absorption peak intensity at 415 nm following BrGO–Cu treatment compared to Cu^2+^, indicating H_2_O_2_ generation. DFT calculations further confirmed the reaction as spontaneous (Δ*E* = −903 kJ mol^−1^) at pH < 7 (Δ*E* = 926 kJ mol^−1^ at alkaline pH, Table S3). The above results demonstrated that the BrGO–Cu bioreactor utilized BEET and GSH in the BME to synergistically promote the conversion of Cu^2+^ to Cu^+^ and enabled H_2_O_2_ self-generation. Furthermore, the ESR signal of 5,5-dimethyl-1-pyrroline *N*-oxide (DMPO)-OH with an amplitude ratio of 1 : 2 : 2 : 1 was significantly intensified in the BrGO–Cu bioreactor, indicating that the BrGO–Cu bioreactor is more favorable for ˙OH generation (Fig. 5g and S22).^45^ As the vitality of bacteria decreased, the bacterial current diminished (Fig. S23), and the pH in the microenvironment increased from 5.5 to 7.4. As a result, ˙OH production is suppressed causing an automatic halt in antimicrobial performance (Fig. 5h). Briefly, in the BrGO–Cu bioreactor, the BEET-driven valence modulation cascade reaction involves O_2_˙^−^ scavenging and GSH depletion, resulting in self-supplied H_2_O_2_ and enhanced ˙OH generation to effectively inhibit biofilm formation (Fig. S24).

### *In vivo* treatment of *S. epidermidis*-infected subcutaneous abscess healing

**Fig. 6:**
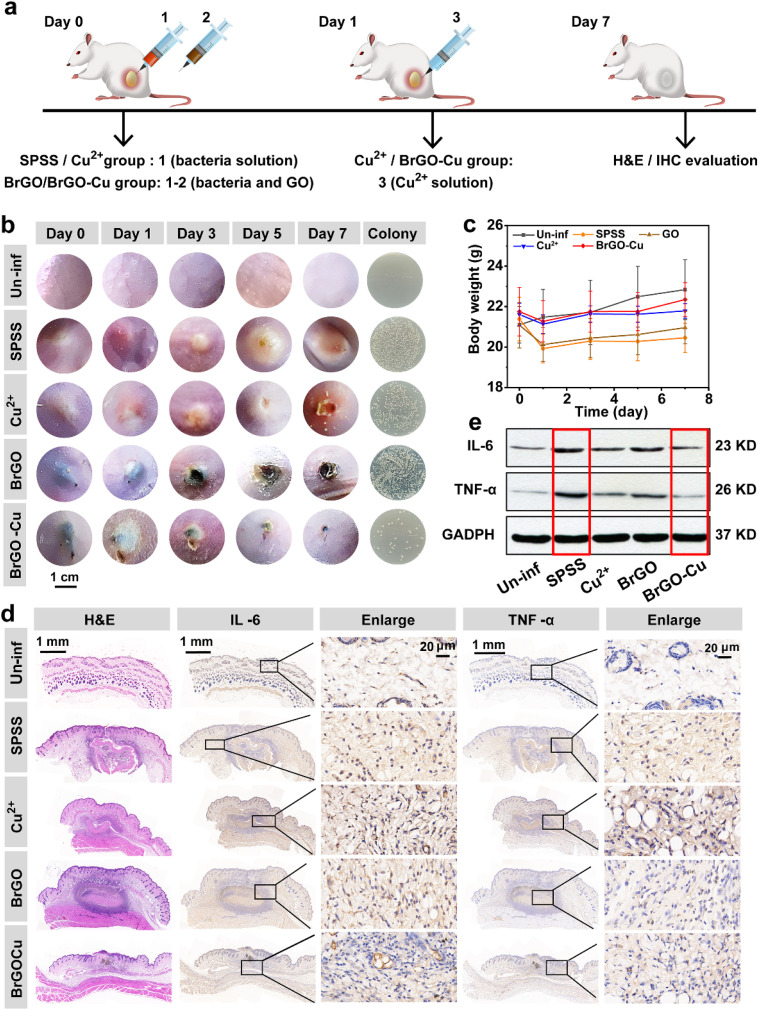
(a) Schematic diagram of subcutaneous abscess formation and the treatment process. (b) Representative photographs of subcutaneous abscess at designated days in stroke-physiological saline solution (SPSS), GO (125 μg mL^−1^), Cu^2+^ (100 μM), and BrGO–Cu (containing 125 μg mL^−1^ GO and 100 μM Cu^2+^) groups (column 6: digital photograph of *S. epidermidis* colonies from infected tissues on day 7). (c) Body weight changes corresponding to (b). (d) H&E and IHC staining images of infected tissues after various treatments on day 7. (e) Protein expression level of IL-6 and TNF-α in abscess tissues by western blotting.

Encouraged by BrGO–Cu's excellent antibacterial ability and biocompatibility *in vitro* through the BEET-driven strategy, we successfully applied BrGO–Cu to treat subcutaneous abscesses induced by *S. epidermidis in vivo*. All experimental mice were randomly divided into five groups (Fig. 6a). The Un-infected (Un-inf) group was a blank control group without any treatment. The infected mice were divided into four groups for different treatments: stroke-physiological saline solution (SPSS), GO, Cu^2+^, and BrGO–Cu. Fig. 6b shows the photographs of abscesses with different treatments on days 0, 1, 3, 5, and 7. After treatment with BrGO–Cu for 5 days, the scars appeared, and the abscess area became generally smaller. In contrast, the cutaneous abscesses in control and other treatment groups healed slowly with evident dermonecrosis and white lesions (filled with fluid/pus). We also visualized bacterial populations around the abscess in each group. The smallest number of colonies belonged to the BrGO–Cu treated group. Moreover, the weight of mice in the BrGO–Cu group recovered faster than the other groups (Fig. 6c), proving that BrGO–Cu offered the best and fastest recovery from *S. epidermidis*-infected abscesses.

In addition, the infected tissues were collected and stained with haematoxylin–eosin (H&E), immunohistochemistry (IHC) for IL-6 and TNF-α to study the anti-biofilm ability of BrGO–Cu. H&E staining showed that the BrGO–Cu group contained the fewest neutrophils compared to the Un-inf group, forming new capillaries. Conversely, other groups' abscesses manifested extensive necrotic polymorphonuclear leukocytes and a significant quantity of neutrophils, suggesting a severe bacterial infection (Fig. 6d, H&E). More importantly, the bacterial infection process was accompanied by changes in inflammatory factors. We then assessed expression levels of IL-6 and TNF-α in the infected tissues by IHC and western blot (WB) (Fig. 6d, IHC and 6e). Infected abscess tissue in the SPSS group secreted a large number of IL-6 and TNF-α compared to the Un-inf group to aggravate the inflammatory response in mice. The BrGO–Cu group was the only infected group that witnessed an inflammation reduction. Meanwhile, the protein expression level of TNF-α (which is an indicator for inflammation response) in the BrGO–Cu group was significantly lower than that in the other groups. The expression of IL-6 also returned to near-normal levels in the BrGO–Cu group, indicating that BrGO–Cu effectively inhibited the inflammatory response (Fig. S25). In brief, the BrGO–Cu bioreactor enhances the catalytic antibacterial ability on demand *via* the BEET-driven valence self-regulating strategy, dynamically inhibits the further development of a subcutaneous abscess, reduces the stimulation of bacterial inflammation, and shows a great anti-infective treatment effect.

## Conclusions

In summary, we successfully assembled a bio-electroactive BrGO–Cu bioreactor exploiting the bacteria-specific BEET process, achieving on-demand bacteria-responsive sterilization with negligible side effects and drug resistance. The BrGO–Cu bioreactor not only traps bacteria to prevent the diffusion of free bacteria, but also fuels the following antibacterial behavior with bacteria's own extracellular electrons (BEET) and delocalized π electrons. The BEET activated the conversion of Cu^2+^ to Cu^+^ for enhanced ˙OH generation, efficiently inhibiting the formation of the bacterial biofilm. Importantly, BrGO–Cu's antibacterial behavior is activated only by live bacteria, allowing prolonged sterilization and preventing bacterial resistance for both Gram-positive and negative bacteria. This new “two birds with one stone” strategy: (1) treats bacterium itself as the stimulus achieving high specificity and thus low toxicity; (2) and powers the antibacterial process using bacteria's own extracellular electrons improving antibacterial efficacy and thus low drug resistance. Despite its promise, the BrGO–Cu's performance remains dependent on the accessibility and redox activity of copper sites, which may diminish over time due to surface fouling or Cu^+^ reoxidation. Future work will focus on enhancing material durability and broadening applicability to more complex microbial environments. Altogether, this BEET-activated, self-sustained sterilization approach offers a promising framework for designing bacteria-responsive materials targeting persistent and biofilm-associated infections.

## Ethical statement

All animal procedures were approved by the Ethics Committee of Wenzhou Institute, University of Chinese Academy of Sciences (approval no. WIUCAS22031403). Six-week-old male BALB/c mice were obtained from the Animal Care and Use Committee of the same institute.

## Author contributions

Mingming Qin, Qiuping Qian, and Yunlong Zhou conceived the project. Mingming Qin and Qiuping Qian synthesized the samples, performed the characterization, and analyzed the data. Feng Jia conducted additional bacteria-related experiments during the revision. Tianxi Shen prepared the schematic illustration of the proposed mechanism. Mingming Qin drafted the initial manuscript. Xiaoqing Gao, Min Wu, Kelong Fan, and Yunlong Zhou contributed valuable experimental insights. Qiuping Qian and Yunlong Zhou supervised the overall project. All authors discussed the results and approved the final version of the manuscript.

## Conflicts of interest

There are no conflicts to declare.

## Supplementary Material

SC-OLF-D5SC04234H-s001

## Data Availability

The data supporting this article have been included as part of the SI. All experimental procedures and supporting data are provided in the SI, including the synthesis and characterization of GO, rGO, and BrGO–Cu; AFM, TEM, FTIR, UV–vis, and XPS analyses; electrochemical measurements; DFT calculations; antibacterial and antibiofilm assays; hemolysis testing; ROS detection; and western blot analysis of inflammatory markers. Supplementary information is available. See DOI: https://doi.org/10.1039/d5sc04234h.
