# Supplementary material for: Bacteria-driven bio-electroactive sterilization
Source: Chem Sci. 2025 Jul 31;16(35):16158–67. doi: 10.1039/d5sc04234h (PMC12337390; doi:10.1039/d5sc04234h)
Supplement: SC-016-D5SC04234H-s001 [file SC-016-D5SC04234H-s001.pdf]

Supplementary Information (SI) for Chemical Science.
This journal is © The Royal Society of Chemistry 2025

Supplementary Information

Bacteria-Driven Bio-Electroactive Sterilization

Mingming Qin [a], Qiuping Qian, [a, *] Xiaoqing Gao, [a] Tianxi Shen, [b] Feng Jia, [a] Min Wu, [a] Kelong Fan [c] and Yunlong Zhou [a, *]

[a*] Zhejiang Engineering Research Center for Tissue Repair Materials, Wenzhou Institute, University of Chinese Academy of Sciences, Wenzhou 325001, P. R. China, zhouyl@ucas.ac.cn, qianqp@ucas.ac.cn

[b] Department of stomatology, Nanchang People's hospital (The Third Hospital of Nanchang), Nanchang 330009, Jiangxi, P. R. China.

[c] CAS Engineering Laboratory for Nanozyme, Key Laboratory of Biomacromolecules (CAS), CAS Center for Excellence in Biomacromolecules, Institute of Biophysics, Chinese Academy of Sciences, Beijing 100101, P. R. China.

Table of Contents

Experimental Procedures **3**

Chemicals and Reagents 3

Synthesis of graphene oxide (GO) solution 3

Synthesis of reduced graphene oxide (rGO) solution 3

Synthesis of BrGO–Cu bioreactor 3

Characterization 4

The bacterial adhesion ability of GO and rGO nanosheets 4

Copper ion concentration in BrGO-Cu composites 4

Distribution of copper elements in BrGO-Cu composites 5

Fenton-like catalytic capability assessment 5

Mechanism of rGO-enhanced BrGO-Cu catalysis 5

Viable bacteria trigger enhanced Fenton-like mechanism 6

Bactericidal activity 7

In Vitro inhibit biofilm formation ability 7

Bacteria-responsive antibacterial of BrGO–Cu bioreactor 8

Cyclic voltammetry test 8

Bacteria current and Impedance Spectroscopy (EIS) detection 9

ESR measurement 9

Hemolysis assay 9

Cell toxicity evaluation 9

Animal experiment 10

Western blot analysis 10

Results **11**

References **21**

Experimental Procedures

Chemicals and Reagents

Sodium nitrate ($NaNO_3$), sulfuric acid (H_2SO_4), potassium permanganate ($KMnO_4$), hydrogen peroxide (H_2O_2, 30%), acetone, and $CuCl_2$ (cupric chloride) were obtained from Sinopharm Chemical Reagent Co., Ltd. Graphite was purchased from Aladdin Holdings Group Co., Ltd. Anhydrous ethanol was obtained from Shanghai Bohr Chemical Reagent Co., Ltd. Sodium acetate (NaAc) and acetic acid (HAc) were obtained from Shanghai Macklin Biochemical Co., Ltd. Dimethyl sulfoxide (DMSO) was purchased from Shanghai Acmec Biochemical Co., Ltd. TMB (3,3',5,5'-tetramethylbenzidine), Luria–Bertani (LB) medium, and Cell Counting Kit-8 (CCK-8) were purchased from the Beyotime Institute of Biotechnology, China. IL-6 and TNF-α antibodies were obtained from Abcam. Deionized water (ddH_2O, 18.2 MΩ·cm) was obtained from a Milli-Q water purification system. All other chemicals were used without further purification.

Synthesis of graphene oxide (GO) solution

Firstly, 2 g of 325-mesh graphite, $NaNO_3$, and 96 mL of H_2SO_4 were stirred in an ice bath. Secondly, 12 g of $KMnO_4$ was slowly added under constant stirring. Secondly, 12 g of $KMnO_4$ was slowly added under constant stirring, maintaining the temperature at 0 °C throughout the addition. The mixture was stirred at 0 °C for 90 min, then transferred to a 35 °C water bath and stirred for an additional 2 h to form a thick paste. Thirdly, 80 mL of distilled water was slowly added dropwise over 40 minutes. Next, 200 mL of distilled water was added, followed by the slow addition of 10 mL of 30% H_2O_2. The solution color changed from dark brown to yellow due to the reduction of residual permanganate (MnO_4^-) and manganese dioxide (MnO_2) to manganese (II) ions (Mn^{2+}) by hydrogen peroxide. Finally, the mixture was subjected to high-speed centrifugation at 10,000 rpm for 8–15 minutes and repeatedly washed with distilled water until the pH approached 7. The resulting precipitate was re-dispersed in water using an ultrasonic cleaner to obtain a GO solution.

Synthesis of reduced graphene oxide (rGO) solution

The GO solution was prepared according to the above method. Then, the GO solution was treated under high temperature (121 °C) and high pressure (100 kPa) for 20 min to obtain the rGO solution.

Synthesis of BrGO–Cu bioreactor

Escherichia coli (*E. coli*, ATCC 8739) and *Staphylococcus epidermidis* (*S. epidermidis*, ATCC 12228) were cultured overnight at 37 °C in Luria–Bertani (LB) broth and tryptone soy broth (TSB), respectively, with shaking at 240 rpm, and then diluted to ~1×10^6 colony-forming units (CFU)/mL to prepare working suspensions. A 5 mL aliquot of the bacterial suspension was mixed with 5 mL of GO solution (250 µg/mL) and incubated at 37 °C for 12 h. The resulting mixture was centrifuged at 5000 rpm for 5 min, washed three times with deionized water (DIW), and re-dispersed in 5 mL DIW to obtain a suspension of bacteria–reduced GO (BrGO) complexes. Subsequently, 5 mL of $CuCl_2$ solution (200 µM Cu^{2+}) was added to the BrGO suspension and mixed thoroughly to yield the BrGO–Cu bioreactor.

Characterization

The UV-vis-NIR Spectrophotometer (CARY5000) analyzed the GO, rGO solution. Scanning electron microscopy (SEM, SU8010, Hitachi) and Transmission electron microscopy (TEM, 200 kV, FEI Talos) were used to observe the morphology of GO, rGO, BrGO, bacteria, and bacterial biofilm. X-ray photoelectron spectroscopy (XPS, K-Alpha, Thermo Fisher Scientific) was used to characterize GO, rGO, BrGO, bacteria–Cu^{2+}, GO–Cu^{2+}, rGO–Cu^{2+}, and BrGO–Cu composites. Raman spectra were recorded using a Raman spectrometer (Renishaw inVia). The concentrations of copper in solution were determined using inductively coupled plasma mass spectrometry (ICP-MS). A full-wavelength microplate reader (Thermo Fisher Varioskan LUX) was used to evaluate the antibacterial and antibiofilm properties of GO, Cu^{2+}, and BrGO–Cu.

The bacterial adhesion ability of GO and rGO nanosheets

QCM-D detection. The GO solution with a mass of 2 mg (1 mg/mL) was uniformly drop-plated onto the surface of Au QCM crystals and dried to obtain GO film–coated Au QCM crystals. *E. coli* or *S. epidermidis* (~10^6 CFU/mL) was added to the QCM cell, and Δf was measured according to the manufacturer's instructions.

Copper ion concentration in BrGO-Cu composites

ICP-MS detection. GO suspension (2 mL, 250 µg/mL) or SPSS (2 mL, 0.9% w/w) was co-incubated with 2 mL of bacterial suspension (1×10^6 CFU/mL; *E. coli* in 1× LB, *S. epidermidis* in 1× TSB) for 12 h, followed by three washes with DIW. Cu^{2+} solution (4 mL, 100 µM) was then introduced to the sediment for 12 h at 37 °C. After reaction, the sample was centrifuged at 10,000 rpm for 5 min and washed with DIW three times to remove weakly bound and unbound copper ions. The supernatant from each centrifugation step was collected and filtered using a 0.22 µm syringe filter. The Cu^{2+} concentration in the filtrate was determined using ICP-MS (Agilent 7700x/7700s, USA). The adsorption rate was calculated using the following equation:

$$\text{Adsorption rate } (\%) = \frac{C_0 - C_{Sample}}{C_0} \times 100 \quad (1)$$

(C_0: 100 μM)

Distribution of copper elements in BrGO-Cu composites

The BrGO–Cu composite was synthesized following the established method and washed three times with DIW to remove weakly bound copper ions. Since BrGO–Cu contains bacteria, the sample was fixed with 2.5% glutaraldehyde overnight and then washed three times with SPSS solution. The composite was dehydrated using a graded ethanol series (30%, 50%, 70%, 80%, 90%, and 99.9%, 15 min each). Then, 10 μL of BrGO–Cu composite solution dissolved in 99% ethanol was dropped onto a molybdenum mesh and left standing for 10 min to remove excess liquid. The mesh with the sample was placed upside down on 10 μL of 1% phosphotungstic acid for 1 min. Excess liquid was removed, and the sample was rinsed three times with DIW.

Fenton-like catalytic capability assessment

˙OH oxidation of TMB. The ability of different components to generate hydroxyl radicals (˙OH) was evaluated using TMB as a substrate in the presence of H_2O_2. The absorbance of the oxidized TMB (at 652 nm) was measured after a set reaction time to assess the amount of ˙OH produced. In a typical experiment, 100 μL of NaAc–HAc buffer (pH = 5.5) or 100 μL of HEPES buffer (pH = 7.4) containing TMB (0.832 mM) and H_2O_2 (100 μM) was mixed with various concentrations of GO (0, 62.5, 125, 250, 500 μg/mL), Cu^{2+} (0, 10, 100, 1000 μM), BrGO–Cu ([GO] = 125 μg/mL, [Cu^{2+}] = 100 μM), GO–Cu^{2+} ([GO] = 125 μg/mL, [Cu^{2+}] = 100 μM), or rGO ([rGO] = 125 μg/mL, [Cu^{2+}] = 100 μM). The mixture was incubated at 37 °C with shaking for 4 h in the dark. After centrifugation, the supernatant was collected, and absorbance at 652 nm was recorded using a Thermo Scientific Varioskan LUX multimode microplate reader.

Mechanism of rGO-enhanced BrGO-Cu catalysis

Computer Simulation Methods. All calculations were performed based on M06[1] /BSI level of theory by Gaussian 09 (revision D.01) software package. The BSI denotes a mixed basis set, which uses a 6-311G(d) [2] basis set for non-metal atoms, and SDD[3] base set for the metal atoms. Geometry optimizations were performed via the Berny algorithm[4] until the total energy converged to within 1×10^{-6} Ha, the forces on all particles were less than 0.0025 a.u., the maximum step size was less than 0.01 a.u., and the root means square (RMS) force was less than 0.006 a.u. (Structural optimization was carried out based on Tu et al 's structural model [6]). The visualized molecular orbitals were shown as an iso-surface value of 0.01 a.u. The binding energy of the

copper ion adsorbed onto rGO was calculated by a formula of $\Delta E = E_{rGO\text{-}Cu^{x+}} - E_{rGO} - E_{Cu^{x+}}$, where $E_{rGO\text{-}Cu^{x+}}$, E_{rGO}, and $E_{Cu^{x+}}$ are the total energies of optimized rGO-Cu^{x+} composite, rGO, and copper ion, respectively (x = 1 or 2). The atomic charge analysis was calculated under the atomic dipole moment corrected Hirshfeld population analysis scheme.

Viable bacteria trigger enhanced Fenton-like mechanism

Dissolved oxygen concentration. The catalytic activity of BrGO–Cu in the bacterial system for H_2O_2 decomposition was evaluated at 25 °C by measuring the dissolved O_2 concentration (unit: mg/L) using a specific oxygen electrode connected to a Multi-Parameter Analyzer (JPSJ-606L, China). In a typical experiment, 150 µL of GO solutions at various concentrations (125, 250, 500, and 1000 µg/mL) were incubated with approximately 10^6 CFU/mL bacteria (150 µL; *E. coli* in 1× LB or *S. epidermidis* in 1× TSB) for 12 hours. After incubation, the reaction was initiated by sequentially adding the BrGO solution and 300 µL of H_2O_2 (final concentration: 1 mM) to 2.4 mL of 0.1 M sodium acetate buffer (pH 5.5).

$O_2^{\cdot-}$ scavenging experiment. The $O_2^{\cdot-}$ scavenging capacity of GO (125 µg/mL), Cu^{2+} (100 µM), and BrGO–Cu ([GO] = 125 µg/mL, [Cu^{2+}] = 100 µM) was evaluated using a commercial total superoxide dismutase detection kit (WST-8). A 20 µL aliquot of each material was taken and mixed with the detection reagent in sequence according to the kit instructions, protected from light, and incubated at 37 °C for 4 h. Under the same reaction conditions, three blank control groups were set as follows: blank group 1 used SOD buffer instead of the sample; blank group 2 contained neither sample nor reaction starting solution; and blank group 3 used DIW as the sample material. The absorbance of the mixtures was then measured at 450 nm using a microplate reader. The inhibition ratio of $O_2^{\cdot-}$ was quantified as follows:

$$Inhibition\ ratio\ (\%) = \frac{Abs\ [(Blank1 - Blank2)/(sample - blank3)]}{Abs(blank1 - blank2)} \times 100\% \quad (2)$$

GSH depletion measurement. To investigate the ability of different materials to oxidize glutathione (GSH), the GSH concentration was measured using a GSH test kit (KeyGen, KGT006). SPSS (negative control), GO (125, 250, 500, and 1000 µg/mL), Cu^{2+} (100 and 1000 µM), BrGO–Cu^{2+} (125–100, 125–200 µg/mL–µM), and GSH (1 mM) were thoroughly mixed at a 1:1 (v/v) ratio. The mixtures were then treated according to the GSH test kit instructions, protected from light, and incubated at 37 °C for 10 h. After incubation, the samples were centrifuged, and the clear supernatant was collected. GSH levels were then quantified spectrophotometrically at 410 nm, and the loss of GSH was calculated as follows:

$$Loss\ of\ GSH\ (\%) = \frac{Abs(negative - sample)}{Abs(negative)} \times 100\% \quad (3)$$

H_2O_2 self-supply. To investigate the H_2O_2 generation ability of BrGO–Cu and Cu^{2+}, the

H_2O_2 level was measured using an H_2O_2 assay kit (Beijing Solarbio Science & Technology Co., Ltd). Cu^{2+} (1000 μM) and BrGO–Cu (125–1000 μg/mL–μM) were each mixed with GSH (0.1 mM) at a 1:1 (v/v) ratio under pH ~5.5 conditions. After incubation at 37 °C for 12 h, the supernatant was collected for analysis according to the kit instructions. In this assay, the indicator component $Ti(SO_4)_2$ in the detection kit reacts with H_2O_2 to form a straw-yellow titanium peroxide complex, which exhibits a characteristic absorption peak at 415 nm.

Bactericidal activity

The antibacterial activities of GO, rGO, Cu^{2+}, rGO–Cu^{2+}, and BrGO–Cu against *E. coli* and *S. epidermidis* were evaluated by quantifying colony-forming units (CFUs) using the plate counting method. Mid-logarithmic phase bacterial cultures were adjusted to 1×10^6 CFU/mL in stroke-physiological saline solution (SPSS). 100 μL bacterial suspension was added to a 96-well plate, followed by 100 μL of SPSS, GO, rGO, Cu^{2+}, rGO–Cu^{2+} (125 μg/mL-100 μM), or BrGO–Cu (125 μg/mL-100 μM). The mixtures were incubated at 37 °C with shaking at 240 rpm for 2 h. After incubation, bacterial suspensions were serially diluted in SPSS, and 10 μL aliquots were plated onto solid agar and incubated at 37 °C overnight. CFUs were counted to assess bacterial viability. Antibacterial efficacy was further evaluated across a range of concentrations for GO (62.5, 125, 250, 500, 1000 μg/mL), Cu^{2+} (20, 200, 2000 μM), and rGO (125, 250, 500, 1000 μg/mL) using the same procedure.

***In Vitro* inhibit biofilm formation ability**

Mid-log-phase *E. coli* and *S. epidermidis* (1×10^6 CFU/mL) were resuspended in LB or TSB and seeded into 96-well plates (100 μL per well), followed by the addition of 100 μL of GO, rGO, Cu^{2+}, rGO–Cu^{2+} or GO–Cu^{2+}. Plates were incubated at 37 °C for 72 h with half-medium replacement every 24 h. Gradient concentrations of GO, rGO, and Cu^{2+} were tested, while composite groups (rGO–Cu^{2+}, GO–Cu^{2+}, and BrGO–Cu) were evaluated at fixed concentrations (125 μg/mL-100 μM). For the BrGO–Cu, bacteria were first co-cultured with 50 μL GO for 24 h, followed by the addition of 50 μL Cu^{2+} and incubation for another 48 h.

CLSM observation. After treatment, wells were washed three times with DIW, fixed at 55 °C for 1 h, and stained with 0.1% crystal violet (200 μL) at 37 °C for 15 min. Excess dye was removed by washing, and plates were air-dried overnight. Crystal violet retained in the biofilm was solubilized with 30% acetic acid (200 μL), and absorbance was measured at 550 nm using a Varioskan LUX multimode reader. For biofilm imaging, bacteria were seeded into glass-bottom culture dishes and treated under identical conditions. After incubation, biofilms were stained with SYTO 9 and propidium iodide (PI) for 30 min and imaged using confocal laser scanning microscopy.

SEM observation. Clean silicon wafers were placed at the bottom of a 96-well plate, and the bacterial biofilm inhibition experiment was conducted as described above. After

72 h, the wafers were removed, washed twice with SPSS, fixed overnight with 3% glutaraldehyde, and washed three times with SPSS. Bacterial cells were then dehydrated sequentially with 30%, 50%, 70%, 80%, 90%, 95%, and 100% ethanol for 10–15 min each. Finally, the bacterial suspension was added to the silicon wafer for air-drying, and the morphology was observed using SEM (SU8010, Hitachi).

Bacteria-responsive antibacterial of BrGO–Cu bioreactor

The Mid-log-phase bacterial cultures were adjusted to 1×10^4 CFU/mL in LB medium. Inactivated BrGO–Cu (I-BrGO–Cu) was washed with DIW by centrifugation, vacuum-dried, and ground into powder. Defined amounts of the powder were redispersed in DIW to prepare I-BrGO–Cu suspensions at 100 or 1000 µg/mL. For bacteria-responsive antibacterial evaluation, 100 µL of bacterial suspension was co-cultured with 100 µL of I-BrGO–Cu solution for 2 h. Treated suspensions were serially diluted, plated on LB agar, and incubated at 37 °C for colony counting.

To further evaluate the antimicrobial kinetics under BrGO–Cu exposure, *E. coli* and *S. epidermidis* were cultured in LB or TSB medium at 37 °C for 24 h and diluted to $\sim 10^4$ CFU/mL. Bacterial suspensions (1 mL) were treated with BrGO-Cu (125µg/mL-50 µM; 1/2 minimum bactericidal concentration) for varying durations. Bacterial growth was assessed by OD_{600} and colony counting. Relative growth rates were determined from the slope (K) of OD_{600} versus time (T), and generation time was calculated using the equation: Generation = T/ $log_{2}^{10^K}$.

For post-treatment evaluation, bacteria exposed to BrGO–Cu for 24 h were centrifuged, resuspended in Cu^{2+} solution (50 µM, 0.5 mL) and fresh medium (0.5 mL), incubated for 1 h, washed three times with PBS, and autoclaved to obtain pre-inactivated BrGO–Cu. To assess whether BrGO–Cu treatment induces bacterial resistance, BrGO–Cu-treated bacteria (24, 48, and 72 h) were inoculated into fresh medium and cultured for 24 h. The resulting suspensions were diluted to $\sim 10^4$ CFU/mL and co-cultured with pre-inactivated BrGO–Cu for 1.5 h. Aliquots (50 µL) were plated on LB agar and incubated at 37 °C for 18–24 h for colony enumeration. Parallel experiments were conducted in three groups.

Cyclic voltammetry test

Electrochemical measurements were performed using a CHI 660E electrochemical workstation (CH Instruments, China) with a conventional three-electrode setup consisting of a glassy carbon working electrode, an Ag/AgCl reference electrode, and a platinum counter electrode. Four systems were evaluated: (1) Bacteria suspension (*E. coli* and *S. Epidermidis*; $\sim 10^9$ CFU/mL), (2) BrGO–Cu (125 µg/mL-100 µM), (3) Cu^{2+} (100 µM), and (4) GO (125 µg/mL). All solutions were adjusted to pH ~5.5. Prior to each measurement, the electrolyte was purged with argon for 15 min to remove dissolved oxygen.

Bacteria current and Impedance Spectroscopy (EIS) detection

The Current–potential (I–V) curves were recorded using a CHI 660E electrochemical workstation (CH Instruments, China) with 5 mM $K_3[Fe(CN)_6]$ as the redox system.The samples were used as working electrodes, with a platinum wire as the counter electrode and Ag/AgCl as the reference electrode. The potential was scanned from −0.6 V to 0.6 V. The samples tested included GO, BrGO, or BrGO–Cu samples For samples with live bacteria, 100 μL of a suspension containing material and bacteria (~10^9 CFU/mL) was drop-cast onto the sample surface and dried at 37 °C for 30 min to form a uniform film. All measurements were performed under dark conditions. Electrochemical impedance spectroscopy (EIS) was carried out at an open-circuit potential of 0.2 V, with a frequency range of 0.01–100,000 Hz. Prior to all measurements, the electrolyte was purged with argon for 15 min to remove dissolved oxygen.

ESR measurement

Hydroxyl radical ($^{\cdot}OH$) generation was analyzed using electron spin resonance (ESR) spectroscopy. Four sample groups (200 μL each) were prepared: (1) bacterial suspension, (2) bacterial suspension + GO, (3) bacterial suspension + Cu^{2+}, and (4) BrGO–Cu. Each sample was mixed with H_2O_2 (10 mM) and DMPO (20 μL) as a spin-trapping agent, and the resulting mixture was transferred to the ESR cavity for signal acquisition at selected time points.

Hemolysis assay

The hemolytic ability of Cu^{2+}, GO and GO-Cu^{2+} was evaluated via a hemolysis test. Healthy BALB/c mice blood containing 3.8% sodium citrate was diluted with SPSS at a 1:9 volume ratio. Then, 0.5 mL of the diluted blood was mixed with 0.5 mL of different concentrations of GO (125, 250, 500, 1000 μg/mL in SPSS), Cu^{2+} (200, 2000 μM in SPSS), and GO–Cu^{2+} (250–200, 250–2000 μg/mL–μM in SPSS). SPSS served as the blank group and deionized water (DIW) as the positive control. Samples were incubated at 37 °C for 1 h, followed by centrifugation at 1500 rpm for 5 min. The absorbance of free hemoglobin was measured at 545 nm using a microplate reader. The relative hemolysis percentage was calculated using the following equation:

$$Hemolysis\ (\%) = \frac{Abs(Sample - Blank)}{Abs(Positive - Blank)} \times 100 \quad (4)$$

Cell toxicity evaluation

L929 cells were seeded (density: 10^5 cells/well) in complete culture medium and incubated at 37 °C with 5% CO_2 for 12 h. Cells were then treated with varying

concentrations of GO and Cu^{2+} for 24 h. Cytotoxicity was assessed using the Cell Counting Kit-8 (CCK-8) assay. Optical density (OD) values were measured at 490 nm using a Thermo Scientific Varioskan LUX multimode microplate reader.

Animal experiment

All animal procedures were approved by the Ethics Committee of Wenzhou Institute, University of Chinese Academy of Sciences (Approval No. WIUCAS22031403). Six-week-old male BALB/c mice were obtained from the Animal Care and Use Committee of the same institute.

Infection model construction. 30 BALB/c mice were divided into 5 groups randomly (n = 6). Uninfected control and received no treatment. The remaining four groups were used to establish a subcutaneous abscess infection model. To construct the model, 100 μL of *S. epidermidis* suspension (~10^8 CFU/mL) was injected subcutaneously into the flank of each mouse. In the GO and BrGO–Cu groups, the bacterial suspension was premixed with GO (250 μg/mL) prior to injection. After 24 h, the mice received the following subcutaneous treatments: (1) normal saline (100 μL), (2) Cu^{2+} (100 μL, 100 μM), (3) SPSS (100 μL), and (4) Cu^{2+} (100 μL, 100 μM), corresponding to their group assignments.

Bacterial load quantification. Following treatment, infected tissues were harvested, homogenized in SPSS, and diluted to ~10^4 CFU/mL. A 50 μL aliquot of each homogenate was plated on LB agar and incubated at 37 °C for 12 h for colony enumeration.

Histopathological analysis. After 7 days of treatment, infected tissues were excised and subjected to hematoxylin and eosin (H&E) staining for histological evaluation. Immunohistochemical (IHC) analysis was performed on abscess sections using antibodies against TNF-α and IL-6. Sections were scanned using a Pannoramic MIDI scanner (3DHISTECH, Hungary), and images were analyzed using CaseViewer.

Western blot analysis

After treatment in each group, abscess tissue samples were collected, and the expression of inflammation-related proteins was measured by western blot analysis. The tissue blocks were homogenized, and cells were collected and washed three times with PBS. The samples were then lysed in PMSF-containing lysis buffer on ice for 30 min. Lysates were centrifuged at 12,000 rpm at 4 °C for 5 min, and the resulting supernatants were collected for analysis. Western blotting was performed according to standard protocols, and protein bands were analyzed using ImageJ software.

Results

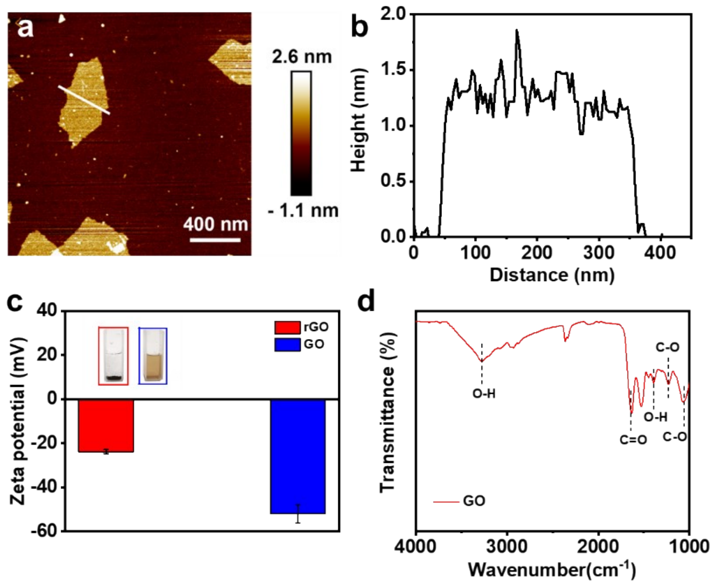

Figure S1. (a) AFM height image of GO films and the corresponding height profile **(b)**. **(c)** Zeta potential of GO and rGO. **(d)** FTIR spectra of GO.

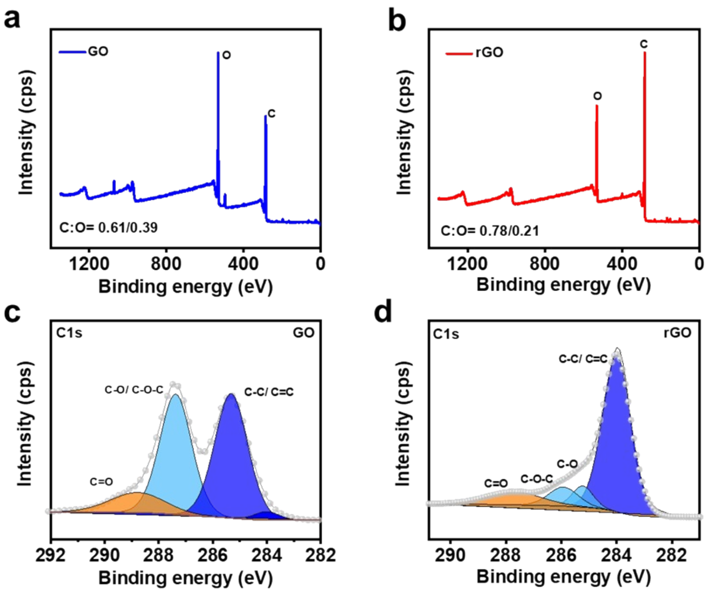

Figure S2. (a) XPS spectrum of GO. **(b)** XPS spectrum of rGO. **(c)** High-resolution XPS spectrum of C 1s in GO. **(d)** High-resolution XPS spectrum of C 1s in rGO.

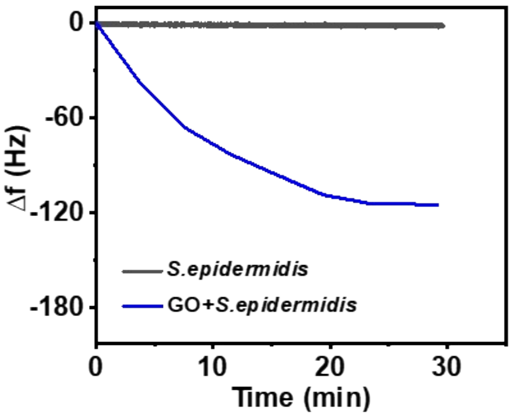

Figure S3. Typical frequency response curve of *S. epidermidis* in the QCM chamber.

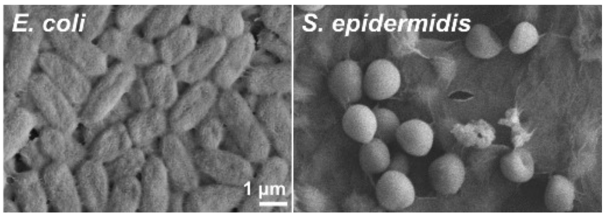

Figure S4. SEM image of bacteria adhered to GO.

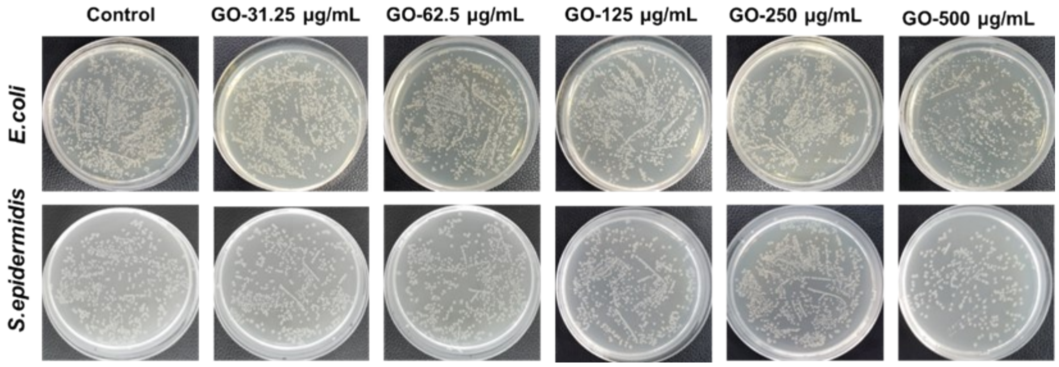

Figure S5. Colony counting to evaluate inhibition of bacterial growth by GO at different concentrations. The surface oxygen content (SOC) of graphene materials dictates their bacterial interaction mode. In suspension, graphene oxide with high SOC predominantly adheres parallel to bacterial surfaces. At the critical SOC threshold of 0.3, interaction shifts to a perpendicular mode. The synthesized graphene oxide (SOC = 0.4) favors planar adhesion, minimizing mechanical damage to bacteria.

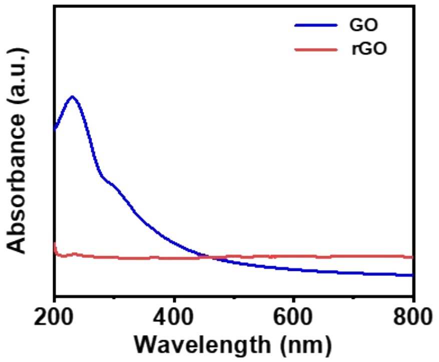

Figure S6. UV-vis absorbance spectra of GO and rGO. The rGO was synthesized by high-temperature and high-pressure methods. The shoulder peak attributed to the n-π* transition of C=O around 310 nm found in GO almost disappears in rGO. The disappearance of oxygen-containing functional groups leads to poor water solubility for rGO.

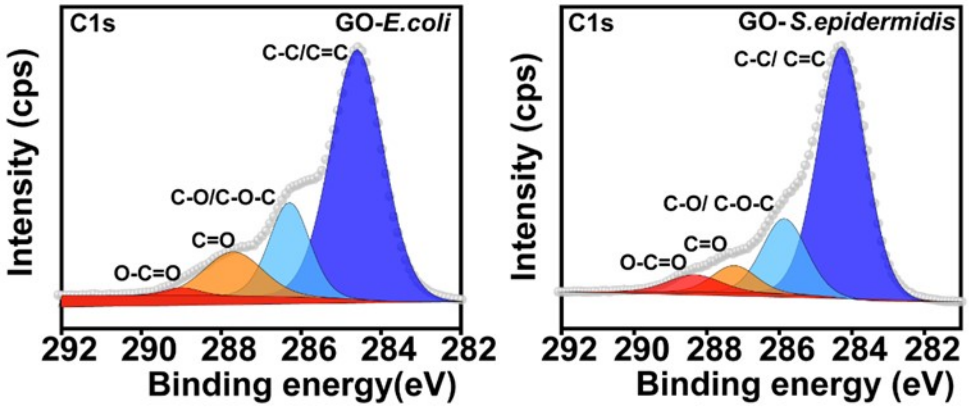

Figure S7. High-resolution XPS spectra for C1s for GO-*E. coli* and GO-*S. epidermidis*.

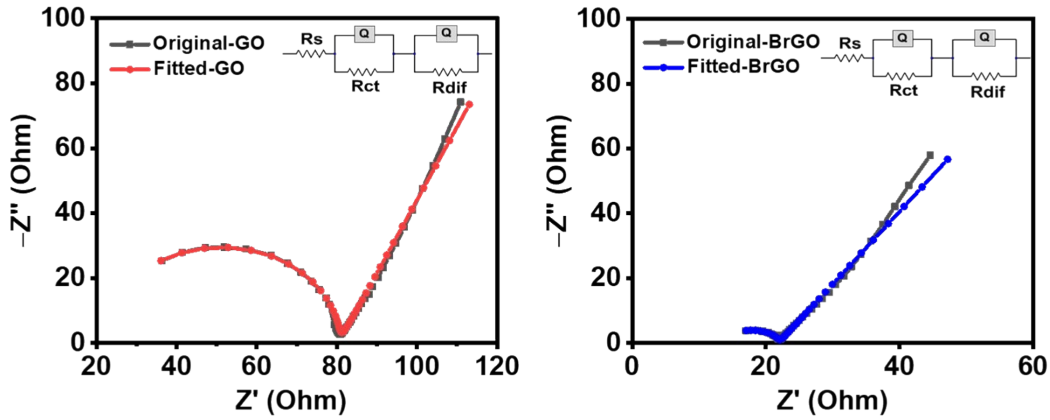

Figure S8. EIS of GO and BrGO (live bacteria and GO were co-cultured for 12 h).

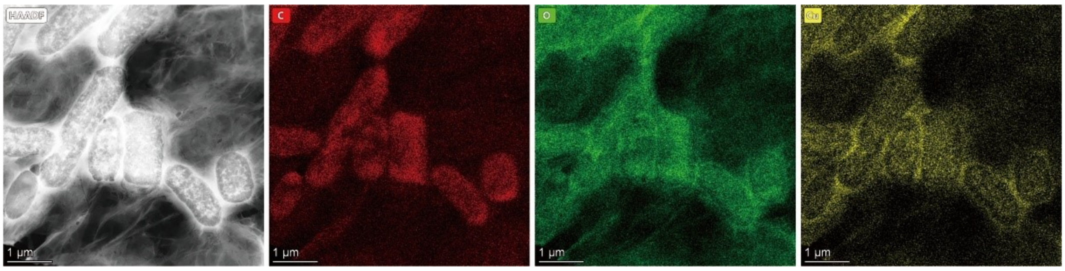

Figure S9. EDS mapping images of BrGO-Cu bioreactor.

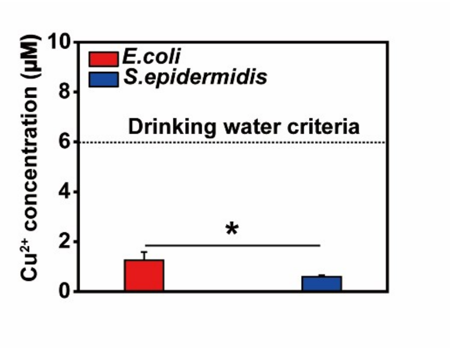

Figure S10. The surrounding copper ions concentration in BrGO-Cu bioreactor solutions.

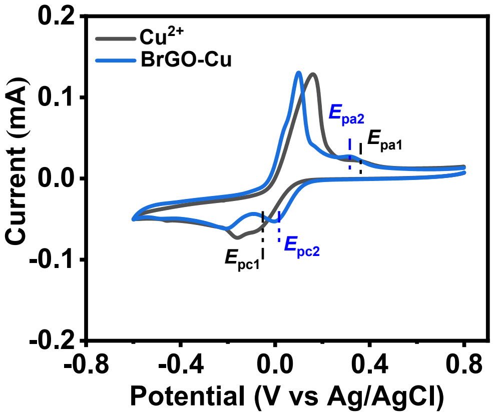

Figure S11. CV curves investigating the electrochemical behavior and redox capability of Cu^{2+} in NaAc-HAc (pH 5.5) with or without the addition of BrGO.

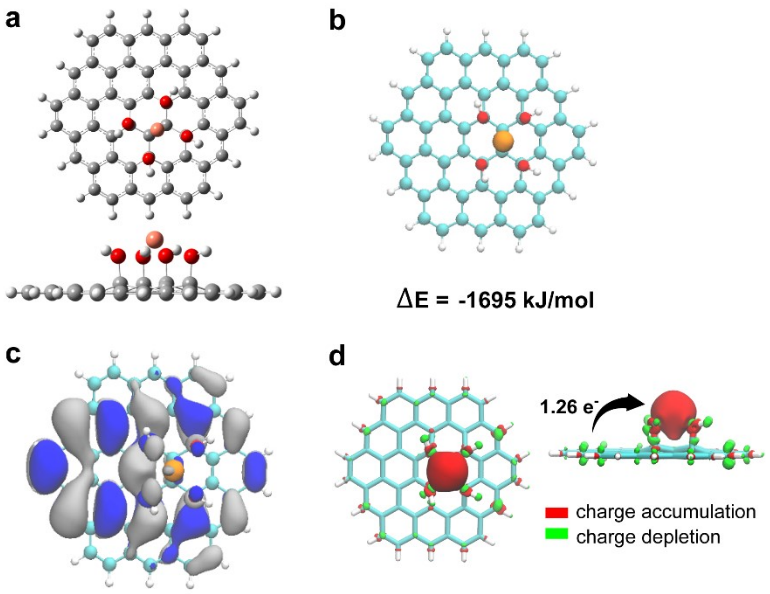

Figure S12. Optimized structures (a) and (b) Binding energy of optimized rGO-Cu^{2+} composite. (C: cyan, O: red, Cu: orange, and H: white). (c) The structure's highest occupied molecular orbital (HOMO) was plotted with an isovalue of 0.01. (Positive phases of wavefunctions: grey region, negative phases of the wavefunctions: blue region). (d) The charge density difference (ρdiff) maps of rGO-Cu^{2+} composite.

The optimized structural model (rGO-Cu^{2+}) of chemical interaction in BrGO-Cu is shown in **Figure S12a**. The binding energy of rGO-Cu^{2+} for Cu^{2+} and rGO was the lowest value (−1695 KJ/mol), suggesting that the rGO-Cu^{2+} system existed most stable (**Figure S12b**). Furthermore, the fundamental rationales for the stable binding of rGO and Cu^{2+} were analyzed from the perspective of molecular orbital interaction. The d orbital of Cu^{2+} is not fully filled (electronic configuration type: [Ar]3d9), and the electrons of rGO in the π-π aromatic loop are highly delocalized. Therefore, the highest occupied states of the molecular orbitals (HOMO) display the strong coupling between the empty d orbitals of copper ion and delocalized π orbitals of the aromatic structure of the rGO surface (**Figure S12c**). The strong non-covalent interaction between the rGO and Cu^{2+}, namely cationic-π interactions, leads to the stable existence of rGO-Cu^{2+} system. In addition, the strong coupling through cation-π between Cu and rGO also resulted in a noticeable charge transfer from rGO to Cu and Cu^{2+}. The charge density difference maps indicated the presence of the interaction and charge transfer at the interface of rGO-Cu^{2+} and that Cu^{2+} as an electron extractor, can accept partial charges from rGO to increase the charge density (**Figure S12d**). Wavefunction analysis showed the charge transition in rGO-Cu^{2+} was 1.26.

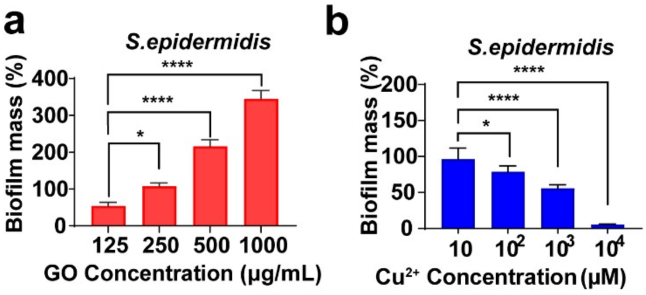

Figure S13. The biofilm mass of *S. epidermidis* was exposed to different concentrations of **(a)** GO and **(b)** Cu^{2+}.

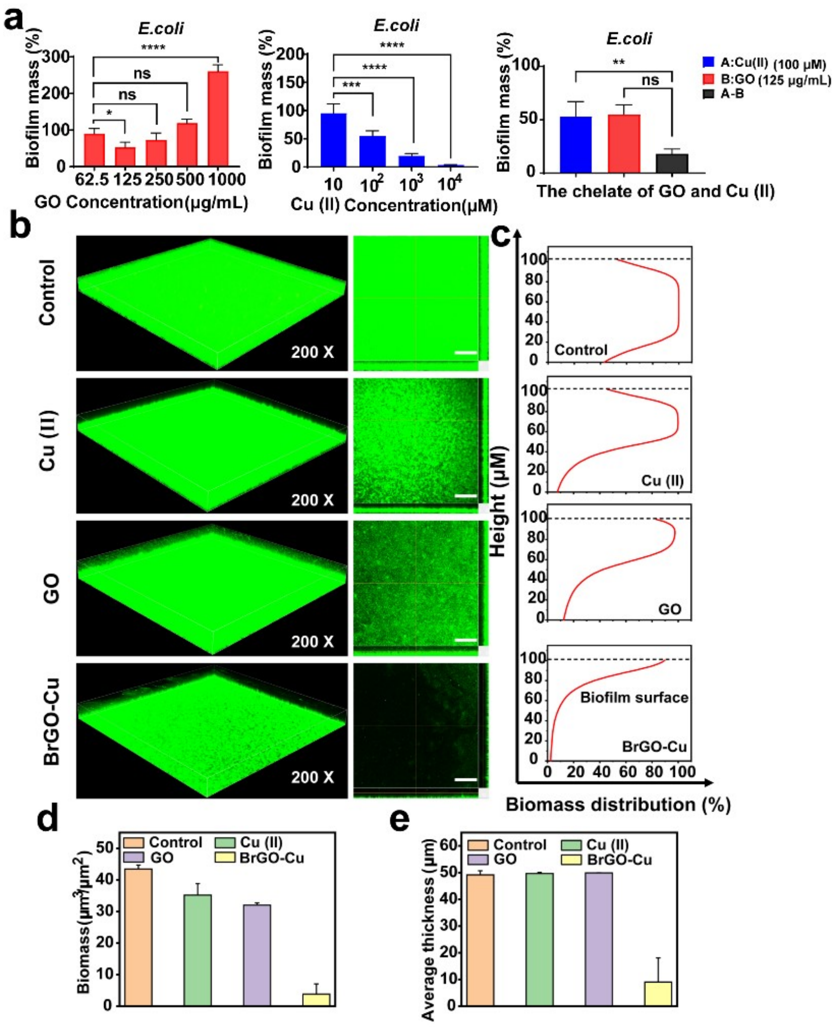

Figure S14. (a) The biofilm mass of *E. coli* was exposed to different concentrations of GO, Cu^{2+} and BrGO-Cu. **(b)** CLSM of preformed *E. coli* biofilms after co-cultivated with Stroke–physiological saline solution (SPSS), GO (125 μg/mL), Cu^{2+} (100 μM) and BrGO-Cu (125 μg/mL-100 μM) composite for 72 h, respectively (green fluorescence: Live-*E. coli* biofilm, Scale bar: 200 μm). The biomass distribution at biofilm height **(c)**, biomass **(d)** and the average thickness **(e)** of *E. coli* biofilms derived from COMSTAT analysis of CLSM images (b).

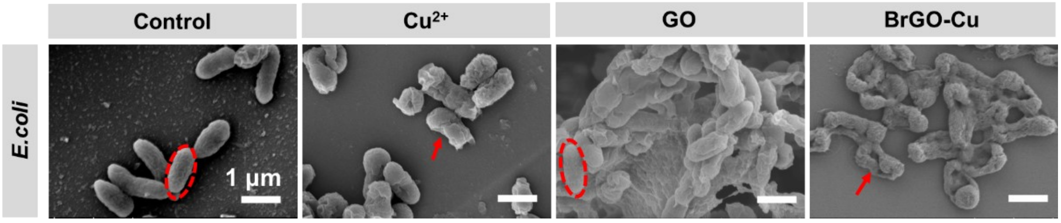

Figure S15. SEM images of *E. coli* after treatment with normal saline (SPSS), Cu^{2+} (100 μM), GO (125 μg/mL), and BrGO-Cu (100 μM-125 μg/mL).

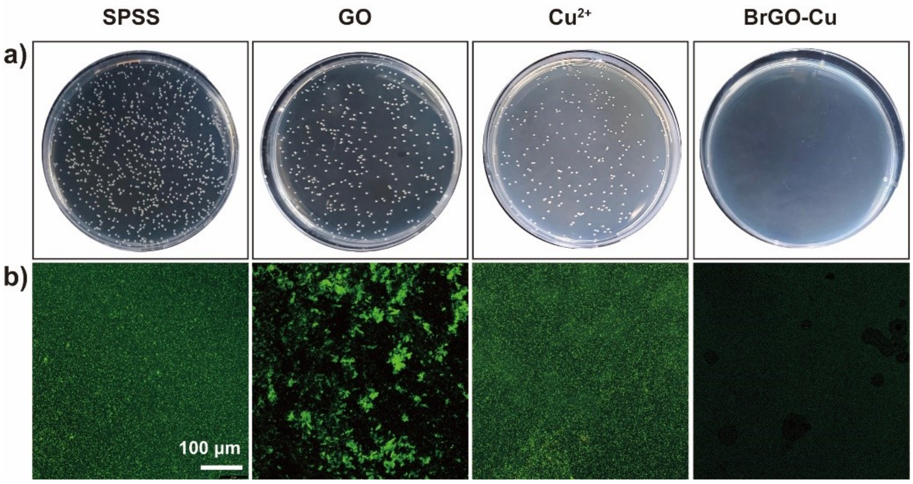

Figure S16. (a) Images of MRSA colonies and (b) biofilm inhibition after treatment with SPSS, GO (125 μg/mL), Cu^{2+} (100 μM), and BrGO–Cu (125 μg/mL–100 μM). Biofilms were visualized by CLSM following SYTO 9/PI staining.

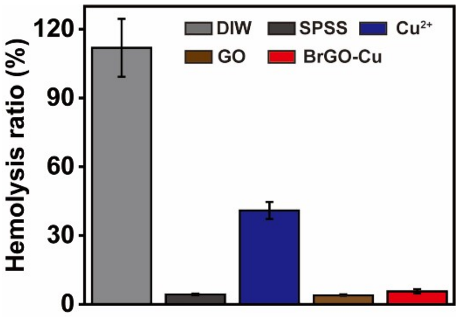

Figure S17. Hemolysis evaluation of DIW, Stroke-physiological saline solution (SPSS), GO (125 μg/mL), Cu^{2+}(100 μM) and BrGO-Cu (125 μg/mL-100 μM).

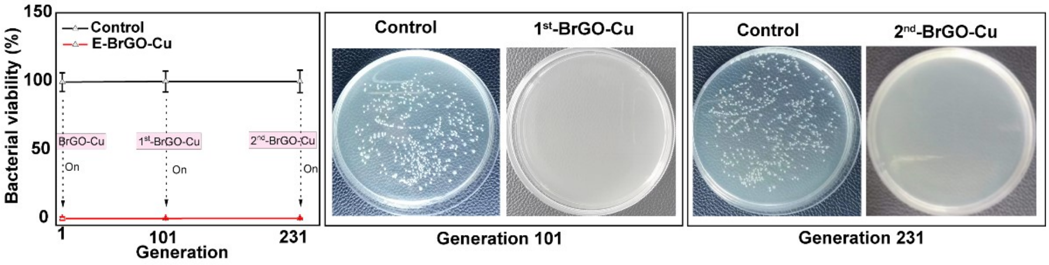

Figure S18. a) Reversible turn-on and turn-off of antibacterial activity based on the

principle of catalytic activity of BrGO-Cu in Living Bacteria Biofilm Environment. b) Colony plating of different generations of *E. coli* treated with SPSS and BrGO-Cu, respectively.

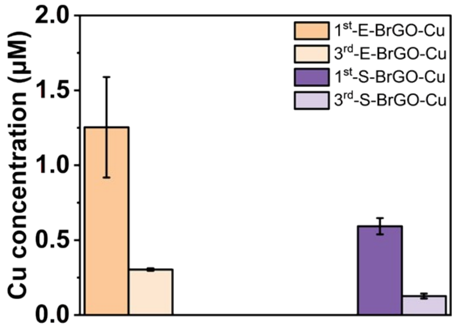

Figure S19. The surrounding copper ions concentrations in BrGO-Cu bioreactor solutions after three sterilization cycles. (E: *E. coli*; S: *S. epidermidis*)

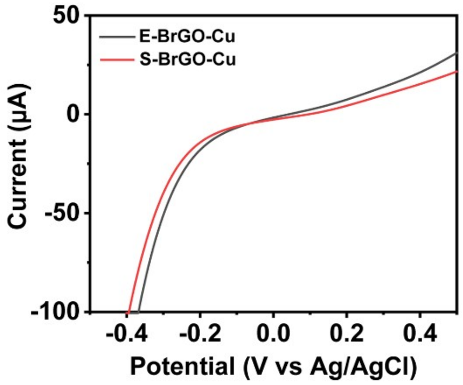

Figure S20. Bacterial Current of BrGO-Cu composite after three sterilization cycles.

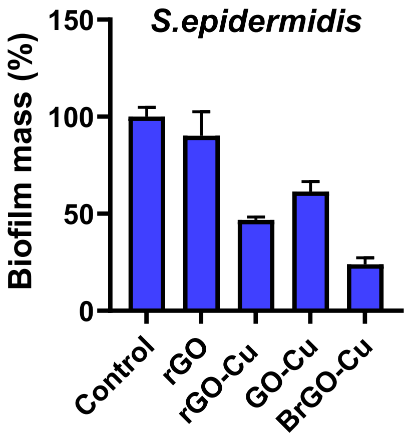

Figure S21. The biofilm mass of *S. epiderimidis* was exposed to GO (125 μg/mL), rGO (125 μg/mL), Cu^{2+} (100 μM), rGO-Cu (100 μM-125 μg/mL), and BrGO-Cu (100 μM-125 μg/mL) composite for 24 h, respectively.

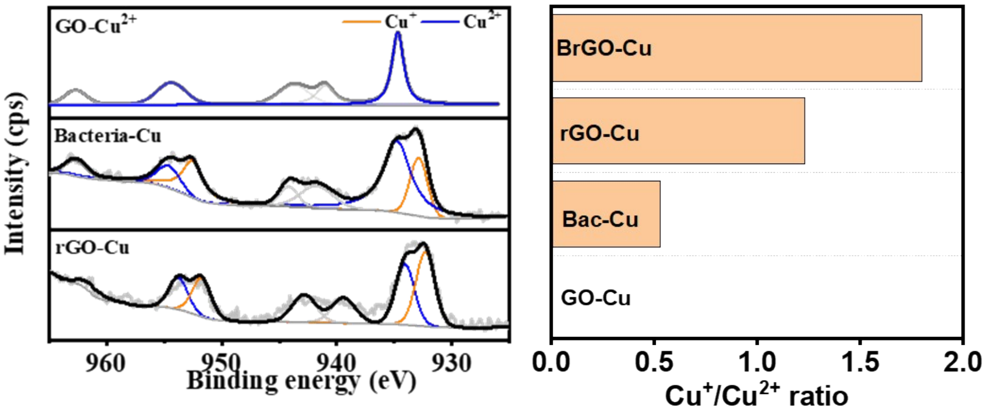

Figure S22. XPS Cu 2p spectra of GO-Cu^{2+}, Bacteria-Cu and rGO-Cu. and Cu^{+}/Cu^{2+} ratios from XPS for BrGO-Cu, rGO-Cu, bacteria-Cu and GO-Cu^{2+}composite.

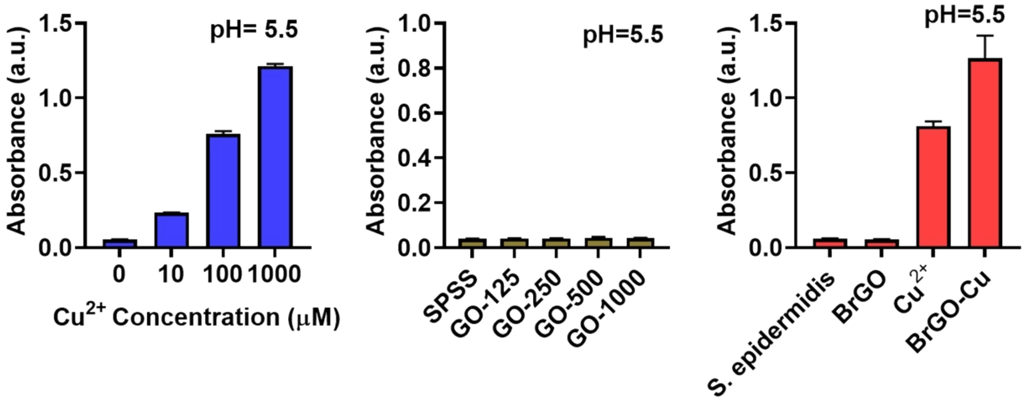

Figure S23. ˙OH generation activity of Cu^{2+}, GO and BrGO-Cu at different concentrations via TMB-based UV-vis spectra at biofilm microenvironment pH~ 5.5 in the presence of H_2O_2 (0.1 mM) (Note: The strains contained in BrGO are *S. epidermidis*).

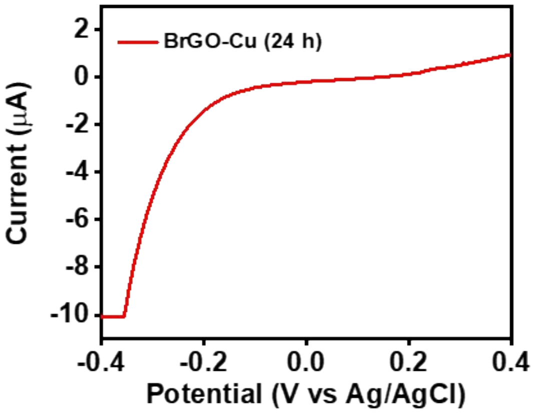

Figure S24. Bacterial Current of BrGO-Cu composite.

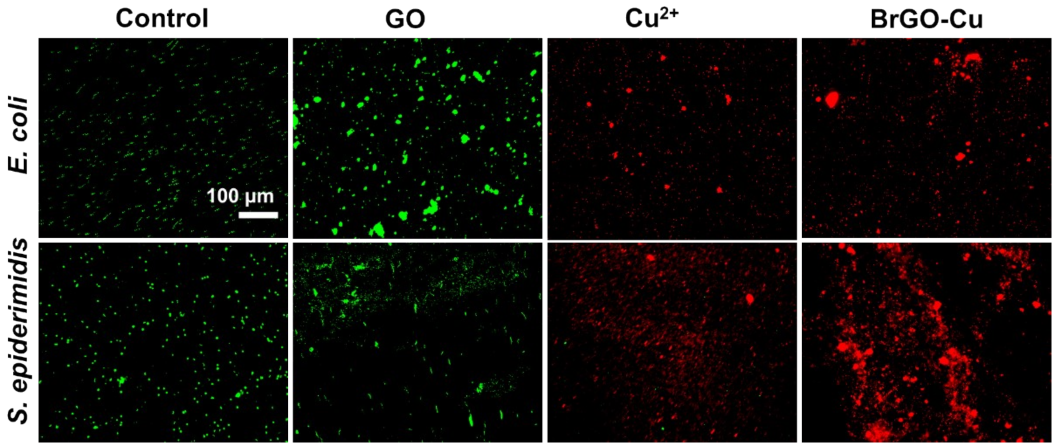

Figure S25. Viability fluorescent staining of *E. coli* and *S. epidermidis* treated with normal saline (SPSS), Cu^{2+} (100 μM), GO (125 μg/mL), and BrGO-Cu (125 μg/mL-100 μM) observed by CLSM.

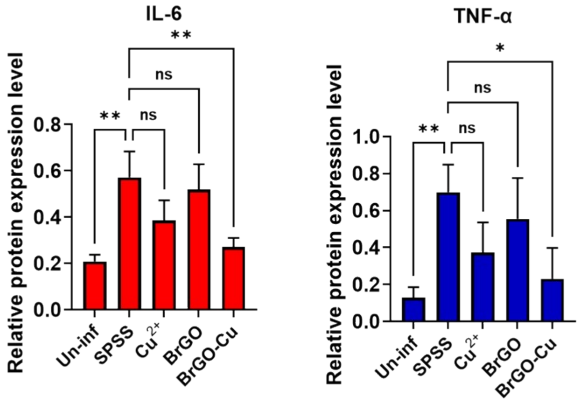

Figure S26. Western blotting quantitative data of IL-6 and TNF-α expression in abscess tissue after different treatments for 7 days.

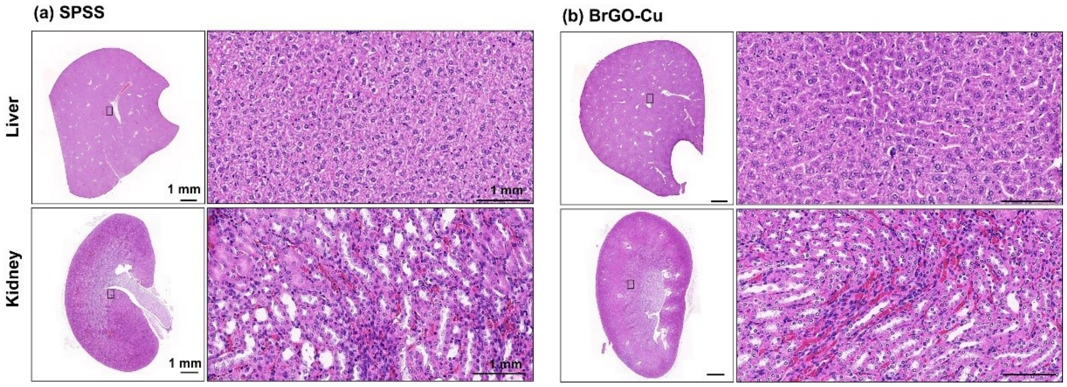

Figure S27. Representative H&E-stained sections of liver and kidney from mice after treatment with (a) SPSS and (b) BrGO–Cu bioreactor.

Table S1. Detailed chemical reactions during CV measurement in **Figure 2g** and **Figure S11**.

Negative scanning	$Cu^{2+} + e^- \rightarrow Cu^+$<br>$Cu^+ + e^- \rightarrow Cu^0$
Positive scanning	$Cu^0 - e^- \rightarrow Cu^+$<br>$Cu^+ - e^- \rightarrow Cu^{2+}$
Negative scanning	$BrGO - Cu^{2+} + e^- \rightarrow BrGO - Cu^+$<br>$BrGO - Cu^+ + e^- \rightarrow BrGO - Cu^0$
Positive scanning	$BrGO - Cu^0 - e^- \rightarrow BrGO - Cu^+$<br>$BrGO - Cu^+ - e^- \rightarrow BrGO - Cu^{2+}$

Table S2. The energy diagram of the reaction process of BrGO-Cu^{x+} adsorbing H_2O_2 and catalyzing the reaction to generate ˙OH

**systems**	**energy barrier (eV)**
rGO-Cu^{2+}	$1.76/2.16^T$
rGO-Cu^+	$1.67/1.34^T$
Cu^{2+}	$4.84/2.87^T$
Cu^+	2.43

with BrGO-Cu^{x+} energy as the reference zero-point, T represents the triplet state, The energy barrier calculation method is the highest value of the energy in the reaction process minus the lowest value.

Table S3. The energy barrier of the reaction process in **Figure 5f**.

Reaction procedure	Energy barrier (KJ/mol)
$BrGO - Cu^{2+} + O^{\bullet -}_2 \rightarrow BrGO - Cu^+ + O_2$	− 903
$BrGO - Cu^+ + \frac{1}{2}O_2 + H^+ \rightarrow BrGO - Cu^{2+} + \frac{1}{2}H_2O_2$	− 781
$BrGO - Cu^+ + \frac{1}{4}O_2 + \frac{1}{2}H_2O \rightarrow BrGO - Cu^{2+} + \frac{1}{2}H_2O_2$	926

References

1. Y. Zhao and D. G. Truhlar, Density functionals with broad applicability in chemistry, *Acc. Chem. Res.*, 2008, **41**, 157-167.
2. A. D. Mclean and G. S. Chandler, Contracted Gaussian-Basis Sets for Molecular Calculations .1. 2nd Row Atoms, Z=11-18, *J. Chem. Phys.*, 1980, **72**, 5639-5648.
3. G. Guzmán-Ramírez, J. Robles, A. Vega and F. Aguilera-Granja, Stability, structural, and magnetic phase diagrams of ternary ferromagnetic 3-transition-metal clusters with five and six atoms, *J. Chem. Phys.,* 2011, **134**, 054101.

4. C. Y. Peng, P. Y. Ayala, H. B. Schlegel and M. J. Frisch, Using redundant internal coordinates to optimize equilibrium geometries and transition states, *J. Comput. Chem.*, 1996, **17**, 49-56.
